# The Impact of Pyrethroid Resistance on the Efficacy of Insecticide-Treated Bed Nets against African Anopheline Mosquitoes: Systematic Review and Meta-Analysis

**DOI:** 10.1371/journal.pmed.1001619

**Published:** 2014-03-18

**Authors:** Clare Strode, Sarah Donegan, Paul Garner, Ahmad Ali Enayati, Janet Hemingway

**Affiliations:** 1 Liverpool School of Tropical Medicine, Liverpool, United Kingdom; 2 Edge Hill University, Ormskirk, United Kingdom; 3 School of Public Health and Health Sciences Research Center, Mazandaran University of Medical Science, Sari, Iran; Kenya Medical Research Institute - Wellcome Trust Research Programme, Kenya

## Abstract

**Background:**

Pyrethroid insecticide-treated bed nets (ITNs) help contribute to reducing malaria deaths in Africa, but their efficacy is threatened by insecticide resistance in some malaria mosquito vectors. We therefore assessed the evidence that resistance is attenuating the effect of ITNs on entomological outcomes.

**Methods and Findings:**

We included laboratory and field studies of African malaria vectors that measured resistance at the time of the study and used World Health Organization–recommended impregnation regimens. We reported mosquito mortality, blood feeding, induced exophily (premature exit of mosquitoes from the hut), deterrence, time to 50% or 95% knock-down, and percentage knock-down at 60 min. Publications were searched from 1 January 1980 to 31 December 2013 using MEDLINE, Cochrane Central Register of Controlled Trials, Science Citation Index Expanded, Social Sciences Citation Index, African Index Medicus, and CAB Abstracts. We stratified studies into three levels of insecticide resistance, and ITNs were compared with untreated bed nets (UTNs) using the risk difference (RD). Heterogeneity was explored visually and statistically. Included were 36 laboratory and 24 field studies, reported in 25 records. Studies tested and reported resistance inconsistently. Based on the meta-analytic results, the difference in mosquito mortality risk for ITNs compared to UTNs was lower in higher resistance categories. However, mortality risk was significantly higher for ITNs compared to UTNs regardless of resistance. For cone tests: low resistance, risk difference (RD) 0.86 (95% CI 0.72 to 1.01); moderate resistance, RD 0.71 (95% CI 0.53 to 0.88); high resistance, RD 0.56 (95% CI 0.17 to 0.95). For tunnel tests: low resistance, RD 0.74 (95% CI 0.61 to 0.87); moderate resistance, RD 0.50 (95% CI 0.40 to 0.60); high resistance, RD 0.39 (95% CI 0.24 to 0.54). For hut studies: low resistance, RD 0.56 (95% CI 0.43 to 0.68); moderate resistance, RD 0.39 (95% CI 0.16 to 0.61); high resistance, RD 0.35 (95% CI 0.27 to 0.43). However, with the exception of the moderate resistance category for tunnel tests, there was extremely high heterogeneity across studies in each resistance category (chi-squared test, *p*<0.00001, *I* ^2^ varied from 95% to 100%).

**Conclusions:**

This meta-analysis found that ITNs are more effective than UTNs regardless of resistance. There appears to be a relationship between resistance and the RD for mosquito mortality in laboratory and field studies. However, the substantive heterogeneity in the studies' results and design may mask the true relationship between resistance and the RD, and the results need to be interpreted with caution. Our analysis suggests the potential for cumulative meta-analysis in entomological trials, but further field research in this area will require specialists in the field to work together to improve the quality of trials, and to standardise designs, assessment, and reporting of both resistance and entomological outcomes.

*Please see later in the article for the Editors' Summary*

## Abstract 2

In a systematic review and meta-analysis, Clare Strode and colleagues assess whether insecticide resistance in African Anopheline mosquitoes affects the efficacy of insecticide-treated bed nets.

*Please see later in the article for the Editors' Summary*

## Editors' Summary

**Background:**

Every year more than 200 million cases of malaria occur worldwide, and more than 600,000 people, mostly children living in sub-Saharan Africa, die from this parasitic infection. Malaria is transmitted to people through the bites of night-flying mosquitoes. Soon after entering the human body, the parasite begins to replicate in red blood cells, bursting out every 2–3 days and infecting more red blood cells. The presence of the parasite in the bloodstream causes malaria's recurring flu-like symptoms, which need to be treated promptly with antimalarial drugs to prevent anemia (a reduction in red blood cell numbers) and life-threatening organ damage. Malaria can be prevented by using insecticides to control the mosquitoes (vectors) that spread the parasite and by sleeping under insecticide-treated bed nets (ITNs) to avoid mosquito bites. High levels of ITN use reduce malaria-related deaths among children by about 20%. Consequently, the widespread provision of ITNs is a mainstay of global efforts to control malaria.

**Why Was This Study Done?:**

About 50% of African households now possess an ITN. However, the emergence of resistance to pyrethroid insecticides—the insecticide class recommended by the World Health Organization for use in ITNs—in some mosquitoes potentially threatens the efficacy of ITNs. Pyrethroids kill *Anopheles* mosquitoes (the main malaria vectors in sub-Saharan Africa) but also prevent mosquitoes entering houses (deterrence), disrupt feeding, and encourage mosquitoes to leave homes prematurely (“induced exophily”; *Anopheles* mosquitoes usually rest inside for a while after feeding). Worryingly, 27 countries in sub-Saharan Africa have already reported resistance to pyrethroids in *Anopheles* mosquitoes. In this systematic review and meta-analysis, the researchers assess the impact of pyrethroid resistance on the efficacy of ITNs against African anopheline mosquitoes in terms of entomological outcomes. A systematic review identifies all the research on a given topic using predefined criteria, meta-analysis uses statistical methods to combine the results of several studies, and entomological outcomes are measures of mosquito behavior and survival.

**What Did the Researchers Do and Find?:**

The researchers identified 25 reports of laboratory and field studies of the impact of ITNs on African malaria vectors that measured the mosquitoes' resistance to pyrethroid insecticides at the time of the study. The laboratory studies used two assays to measure entomological outcomes. The cone test measured mosquito mortality (death), percent of mosquitoes knocked down (immobilized) after 60 minutes, and the time to knock down 50% or 95% of the mosquitoes after brief exposure to an ITN or untreated bed net (UTN). In the tunnel test, mosquitoes had to pass through a holed ITN or UTN to reach animal baits; counts of live and dead mosquitoes, and fed and unfed mosquitoes on both sides of the net measured deterrence, blood feeding, and mosquito mortality. In the field studies, volunteers slept under an ITN or UTN in an experimental hut. Subsequent counts of live and dead mosquitoes and fed and unfed mosquitoes inside the huts and in exit traps measured deterrence, blood feeding, mosquito mortality, and induced exophily. The researchers report that the measurement of insecticide resistance was inconsistent across the identified studies. Nevertheless, their analysis found that ITNs are more effective than UTNs in relation to mosquito mortality, regardless of resistance. There was a relationship between resistance and the risk difference for mosquito mortality in laboratory and field studies, but the substantive variation between studies means that the findings should be interpreted with caution.

**What Do These Findings Mean?:**

These findings show that pyrethroid resistance clearly affects entomological outcomes in laboratory studies, and suggests that this pattern may also be observed in field trials. However, ITNs remained at least somewhat effective despite insecticide resistance in terms of personal protection. The researchers note that there was considerable variability (heterogeneity) among the results obtained in the field trials and suggest that poorly standardized methods and reporting might have masked the true relationship between insecticide resistance and ITN efficacy in these studies. Thus, although ITNs continue to have a substantive effect in many laboratory studies in the face of insecticide resistance, whether ITNs are likely to remain effective against insecticide-resistant mosquitoes in the real world cannot be definitively concluded. Malaria experts and vector biologists need to work together to improve the quality of field trials and to standardize the measurement of insecticide resistance and entomological outcomes, suggest the researchers. Such collaborations, they conclude, are essential to provide the data that policy makers need to plan malaria control strategies.

**Additional Information:**

Please access these websites via the online version of this summary at http://dx.doi.org/10.1371/journal.pmed.1001619.

- Information is available from the World Health Organization on malaria (in several languages); the World Malaria Report 2013 provides details of the current global malaria situation
- Information is available from the World Health Organization on a call for action to tackle the growing threat of insecticide resistance and to facilitate the development of innovative vector control tools and strategies (in English, French and Spanish)
- The US Centers for Disease Control and Prevention provide information on malaria (in English and Spanish) and on insecticide-treated bed nets; it also provides a selection of personal stories about malaria
- Information is available from the Roll Back Malaria Partnership on the global control of malaria and on the Global Malaria Action Plan (in English and French); its website includes fact sheets about malaria in Africa and about insecticide-treated bed nets
- MedlinePlus provides links to additional information on malaria (in English and Spanish)

## Introduction

The World Health Organization (WHO) estimates that there were 655,000 malaria deaths in 2010, with 86% occurring in children under 5 y [1]. Malaria deaths are declining with the massive scaling up of control measures, of which insecticide-treated bed nets (ITNs) are a major component. ITNs reduce deaths in children [2] and provide personal protection to the user, and at scale they provide community-wide protection by reducing the number of infective mosquitoes in the vicinity where ITNs are used [3],[4]. Between 2008 and 2010, 254 million ITNs were supplied to countries in sub-Saharan Africa, and the proportion of African households in possession of a net rose from 3% in 2000 to 50% by 2010 [5]. Nets, when in good condition and used correctly, are effective, simple to use, easy to deliver to rural communities, and cost-effective when used in highly endemic malarious areas [6]. On account of their low mammalian toxicity, speed of action, and high insecticidal activity, pyrethroids [7] are the only insecticide class recommended by the WHO for use in ITNs [8]. ITNs are effective with the African vectors *Anopheles gambiae* s.s. and *An. funestus* in part because these species are endophagic (feed indoors) and endophilic (rest indoors after feeding). Aside from their insecticidal activity, pyrethroids also exert an excito-repellency effect, which can lead to fewer mosquitoes entering a home (deterrence) where ITNs are used, or can cause disrupted blood feeding and premature exit of mosquitoes from the home (induced exophily) [9]. Because of the excito-repellency property of ITNs, these nets retain their personal protection properties for users even after the nets become holed [10].

The emergence and spread of insecticide resistance to all four classes of public health insecticides (pyrethroids, organochlorines, organophosphates, and carbamates) threatens the effectiveness of ITNs and indoor residual house spraying. Currently, 27 countries in sub-Saharan Africa have reported pyrethroid resistance in *Anopheles* vectors [11]. The real figure could very well be higher, as a lack of in-country resistance monitoring prevents accurate assessment. Because of their pyrethroid dependency, ITNs are especially vulnerable to insecticide resistance, as unlike indoor residual house spraying there are no readily available alternative insecticides. To prevent amplifying pyrethroid resistance, the WHO recommends that pyrethroid insecticides should not be used for indoor residual house spraying in areas with high long-lasting insecticide-treated bed net (LLIN) coverage [1]. In a recent study the extensive deployment and use of LLINs was blamed in part for selecting resistance in *Anopheles* vectors in Senegal, where malaria morbidity also increased [12]. The threat of resistance has led the WHO and members of the Roll Back Malaria Partnership to produce the “Global Plan for Insecticide Resistance Management in Malaria Vectors”, which stresses the urgency with which this problem needs to be addressed [13].

Insecticide resistance takes multiple forms: target-site resistance, metabolic resistance, and cuticular resistance. Target-site resistance to pyrethroids in *An. gambiae* and *An. arabiensis* is underpinned by a non-silent point mutation (either L1014F or L1014S) in the sodium channel gene, which is referred to as the knock-down resistance (*kdr*) genotype [14],[15]. Target-site resistance prevents the successful binding of the insecticide molecule to sodium channels on the nerve membranes. Metabolic resistance is caused by the activity of three large multi- gene families (cytochrome P450s, glutathione transferases, and carboxylesterases) that are able to metabolise or sequester the insecticide, thereby preventing it from reaching its target [16]. It is becoming clear that the cytochrome P450s are responsible for the majority of cases of metabolic resistance, with a secondary role for the glutathione transferases [17]–[20]. There is also preliminary evidence that cuticular resistance may be a contributing factor, but this aspect requires further analysis [17],[18],[21]. As pyrethroids and the organochlorine insecticide DDT target the sodium channel protein, cross-resistance to both insecticides is common. There is evidence that phenotypic resistance and *kdr* frequency have increased following the introduction of ITNs in some areas [22],[23], which could nullify the effectiveness of ITNs [24].

Policy makers and researchers debate whether these various forms of resistance are having an impact on the effectiveness of ITNs in malaria control. We carried out a systematic review of all relevant studies on human outcomes, but it became clear very quickly that there was an almost total absence of evidence to draw any conclusions on the impact of pyrethroid resistance on the efficacy of nets in decreasing disease transmission. So we turned to entomological studies: evidence of an effect of resistance on mosquitoes could be indicative of resistance having an impac on disease transmission. Our objective is to assess the effects of insecticide resistance in African anopheline mosquitoes on ITNs in terms of entomological outcomes in precise laboratory assays (cone tests), in laboratory tests with animals (tunnel tests), and in field trials with human volunteers as the attractants.

## Methods

### Inclusion Criteria

#### Study design

We included laboratory tests (cone tests and tunnel tests) and field trials using experimental huts (see Box 1 for details of types of studies included).

**Box 1. Types of Studies Included**

**Cone Test**

**Methods:** Studies in the laboratory in which mosquitoes are placed inside a plastic cone that is attached to a net for three minutes; after net exposure the mosquitoes are placed in a holding container while entomological outcomes are measured [25].

**Outcomes:** Mosquito mortality after 24 h, percentage knock-down at 60 min, and time to 50% or 95% knock-down.

**Advantages:** Researchers can standardise confounding variables, such as mosquito species, sex, age, and blood feeding status. The number of mosquitoes used in the test is standardised.

**Tunnel Test**

**Methods:** Studies in a laboratory, using animal bait, such as a guinea pig, placed at one end of a specially constructed tunnel. A fixed number of mosquitoes are released at the other end of the tunnel, and they must pass through a holed ITN or UTN to reach the animal bait. The following morning, both live and dead mosquitoes, blood fed and non-blood fed, are collected and counted from both sides of the holed net. Live mosquitoes are monitored for a further 24 h to assess delayed mortality [25].

**Outcomes:** Deterrence (not passed through net), blood feeding, and mosquito mortality.

**Advantages:** As for cone test.

**Field Trials**

**Methods:** Studies in areas where mosquitoes breed. Volunteers sleep in experimental huts for a specific period under an ITN or an UTN, with one hut per person. The huts are identical in construction, and incorporate exit traps to catch wild mosquitoes entering and exiting the hut prematurely. Each morning of the trial, both live and dead mosquitoes, blood fed and non-blood fed, are collected and counted from both inside the hut and the exit traps. Live mosquitoes are monitored for a further 24 h to assess delayed mortality. Volunteers and nets are randomly allocated to huts at the start of the trial and are usually rotated to avoid bias. Often huts are cleaned between rotations to avoid cross-contamination of huts from the different treatment arms [25].

**Outcomes:** Deterrence, blood feeding, mosquito mortality, and induced exophily.

**Advantages:** Given that this method assesses the response of wild mosquitoes to human volunteers, it is a more realistic representation of how effective ITNs are in terms of entomological outcomes, compared with laboratory methods.

#### Mosquito population

Included African malaria vectors were *An. gambiae*, *An. arabiensis*, or *An. funestus*. We included laboratory studies that used established laboratory-colonised strains of mosquitoes with known resistance phenotype or genotype. Experimental hut study trials were included if they measured the resistance status of the wild mosquito populations at the time of the study by bioassays with our without *kdr* genotyping.

#### Intervention

We included studies that compared an ITN (conventionally treated bed net [CTN] or a LLIN) versus an untreated bed net (UTN). The CTNs (which require dipping into insecticide and which also require retreatment at least once a year) must have been impregnated with a WHO-recommended pyrethroid with the recommended formulation and dose (see Table 1 for recommended impregnation regimens). The LLINs (which are factory-treated nets where the insecticide is incorporated within or bound around the net fibres) must have had either interim or full recommendation from the WHO (see Table 2 for recommended LLINs).

**Table 1:** WHO-recommended pyrethroids for treatment of CTNs for vector control.

Pyrethroid	Formulation	Dosage^a^
Alpha-cypermethrin	SC 10%	20–40
Cyfluthrin	EW 5%	50
Deltamethrin	SC 1%; WT 25%; WT 25%+binderK-o^b^	15–25
Etofenprox	EW 10%	200
Lambda-cyhalothrin	CS 2.5%	10–15
Permethrin	EC 10%	200–500

a Milligrams of active ingredient per square metre of netting.

b K-O Tab 1-2-3.

CS, capsule suspension; EC, emulsifiable concentrate; EW, emulsion, oil in water; SC, suspension concentrate; WT, water dispersible tablet.

**Table 2:** WHO-recommended LLINs for vector control.

Product Name	Product Type	Status of WHO Recommendation
DawaPlus 2.0	Deltamethrin coated on polyester	Interim
Duranet	Alpha-cypermethrin incorporated into polyethylene	Interim
Interceptor	Alpha-cypermethrin coated on polyester	Full
LifeNet	Deltamethrin incorporated into polypropylene	Interim
MAGNet	Alpha-cypermethrin incorporated into polyethylene	Interim
Netprotect	Deltamethrin incorporated into polypropylene	Interim
Olyset	Permethrin incorporated into polypropylene	Full
OlysetPlus	Permethrin and piperonyl butoxide incorporated into polyethylene	Interim
PermaNet 2.0	Deltamethrin coated on polyester	Full
PermaNet 2.5	Deltamethrin coated on polyester with strengthened border	Interim
PermaNet 3.0	Combination: deltamethrin coated on polyester with strengthened border (side panels) and deltamethrin and piperonyl butoxide incorporated into polyethylene (roof)	Interim
Royal Sentry	Alpha-cypermethrin incorporated into polyethylene	Interim
Yorkool LN	Deltamethrin coated on polyester	Full

#### Outcomes

Included outcomes were blood feeding, mosquito mortality, deterrence (reduction in the number of mosquitoes found in experimental huts), induced exophily (number of mosquitoes found in the exit trap of experimental huts), not passed though net (measure of deterrence in tunnel test), percent knock-down at 60 min, time to 50% knock-down, and time to 95% knock-down [25] (Table 3).

**Table 3:** Measured outcomes appropriate for the different types of study.

Outcome	Description	Laboratory Methods		Field Method: Experimental Hut Trial
		Cone Test	Tunnel Test	
Blood feeding	A measure of the number of mosquitoes that have fed within a hut or in a tunnel during a lab test. Indicates how effective an ITN is in protecting the person sleeping under it (personal protection).		√	√
Mosquito mortality	Measured as the number of mosquitoes killed following exposure to an ITN or UTN, either immediate death or delayed death (24 h following exposure). Measured as a proportion of the total number of mosquitoes found within a hut or placed in tunnel/cone during a lab test. Indicates how effective an ITN is at directly killing mosquitoes.	√	√	√
Induced exophily	Measured as the proportion of mosquitoes found in exit traps, which indicates an attempt to prematurely exit the hut. Indicates how effective an ITN is in protecting the person sleeping under the net (personal protection).			√
Deterrence	A reduction in the number of mosquitoes entering a hut using an ITN relative to the number of mosquitoes found in a control hut using an UTN. Indicates how effective an ITN is in protecting the person sleeping under the net (personal protection).			√
Not pass through net	Equivalent to deterrence in hut trials; measured as the number of mosquitoes that do not pass through a holed ITN to reach an animal bait relative to an UTN in a control test. Indicates the potential effectiveness an ITN could have in protecting the person sleeping under the net.		√	
Knock-down at 60 min	The number of mosquitoes that are knocked down (the inability of a mosquito to fly or stand) within 60 min following exposure to a net.	√		
Time to 50% knock-down	The time taken to knock down 50% of mosquitoes used in the test.	√		
Time to 95% knock-down	The time taken to knock down 95% of mosquitoes used in the test.	√		

### Search Strategy

The search period was from 1 January 1980 to 17 May 2013 or later. We searched the following databases for relevant studies: MEDLINE (from 1 January 1980 to 31 December 2013) and Cochrane Central Register of Controlled Trials, Science Citation Index Expanded, Social Sciences Citation Index, African Index Medicus, and CAB Abstracts (from 1 January 1980 to 17 May 2013). There was no language restriction (see Table S1 for the search terms used).

We also searched the following conference proceedings: First MIM Pan-African Malaria Conference, Senegal, 6–9 January 1997; Second MIM Pan-African Malaria Conference, South Africa, 15–19 March 1999; Third MIM Pan-African Malaria Conference, Tanzania, 17–22 November 2002; Fourth MIM Pan-African Malaria Conference, Cameroon, 13–18 November 2005; Fifth MIM Pan-African Malaria Conference, Nairobi, 2–6 November 2009; American Society of Tropical Medicine and Hygiene 59th Annual Meeting, Atlanta, Georgia, 3–7 November 2010; American Society of Tropical Medicine and Hygiene 60th Annual Meeting, Philadelphia, Pennsylvania, 4–8 December 2011; and American Society of Tropical Medicine and Hygiene 61^st^ Annual Meeting, Atlanta, Georgia, 11–15 November 2012.

### Study Selection

Two authors (C. S. and A. A. E.) independently screened the search results for potentially relevant studies and retrieved the corresponding full articles. C. S. and A. A. E. independently assessed the articles for eligibility using a standardised form (Table S2). Discrepancies between the eligibility results were resolved by discussion. Study investigators were contacted for clarification if the eligibility of a particular study was unclear. Multiple publications from the same study were identified, and if eligible, the original study was taken forward for inclusion.

### Data Extraction

C. S. and A. A. E. independently extracted data from all included studies into a data extraction form. Missing or unclear outcome data were requested from the study investigators. For dichotomous outcomes for the ITN and UTN groups, the number of mosquitoes experiencing the outcome and the total number of mosquitoes were extracted (Tables S3–S5). For continuous outcomes, we extracted the mean and standard deviation when possible. For deterrence, the total number of mosquitoes was extracted for the ITN and UTN groups. A sub-sample of 10% of the studies was randomly selected to assess the performance of the duplicate extraction processes by C. S. and A. A. E. Differences between the two extraction processes were examined, and no serious discrepancies were found. The data extracted by C. S. were used in all analyses.

### Stratification of Resistance

The WHO classifies mosquitoes as susceptible to insecticides if, after exposure to a diagnostic dose, there is ≥98% mortality, and as resistant to insecticides if there is ≤90% mortality; mortality between 97% and 90% requires the confirmation of resistance genes for mosquitoes to be classified as resistant [26]. Characterisation of resistance across studies was not consistent, as some studies used bioassays, others used *kdr* genotyping, and some used a combination of both. We therefore developed a composite classification system to allow us to categorise the insecticide resistance status of mosquitoes in three broad groups (low, moderate, and high), based on phenotypic resistance measured using bioassay mortality data and/or *kdr* frequency (Table 4). The alleles for *kdr* are presented as a frequency or percentage.

**Table 4:** Stratification of mosquito resistance constructed for this study based on either percent mortality from WHO bioassay data and/or *kdr* frequency.

Resistance Status	Percent Bioassay Mortality	*kdr* Frequency (Percent)
**High**	<25 (low mortality)	>80 (high *kdr*)
	<25 (low mortality)	<25 (low *kdr*)
**Moderate**	25–80 (moderate mortality)	25–80 (moderate *kdr*)
	25–80 (moderate mortality)	<25 (low *kdr*)
**Low**	>80 (high mortality)	<25 (low *kdr*)
**Unclear**	<25 (low mortality)	<25 (low *kdr*)

### Risk of Bias Assessment

C. S. assessed the risk of bias of each included study. We developed a quality assessment tool that used four criteria for tunnel and cone tests: (1) comparability of mosquitoes in ITN and UTN groups (all female, age matched, and non-blood fed), (2) observers blinded, (3) complete outcome data, and (4) raw data reported for ITN and UTN groups.

For experimental hut trials we developed seven criteria: (1) comparability of mosquitoes in ITN and UTN huts, (2) collectors blinded, (3) sleepers blinded, (4) raw data reported for ITN and UTN groups, (5) ITNs randomly allocated to huts, (6) ITNs rotated, and (7) sleepers rotated. For all criteria, we made a judgement of high, low, or unclear risk of bias.

For hut trials, we generated an additional set of variables to assess variability in the design and execution of the studies, called “rigor of implementation”. The criteria assessed included (1) nets being washed according to WHO protocol, (2) cleaning of huts before the trial and between rotations to avoid cross-contamination of huts from the different treatment arms and to remove any insects that may have been missed during collections, (3) whether ITNs were tested either chemically or using bioassays to assess the insecticide impregnation efficacy and residual activity (applicable to CTNs), and (4) whether male mosquitoes were excluded from the analysis. We also reported how each study measured resistance in the wild mosquito populations: whether phenotypic resistance was measured by bioassays and/or *kdr* genotyping (and the number of mosquito screened for *kdr*), and whether metabolic resistance was measured.

### Data Analysis

Analyses were carried out in Review Manager 5. We stratified the analyses by study design and the resistance status of the mosquito population (Table 4). Dichotomous outcomes were summarised using the RD; therefore, results are generalisable only to situations where the UTN group event rate is comparable to those observed here. When the same study compared multiple ITNs, the event rate in the UTN group was split to ensure each mosquito was included in the analysis only once.

The results of studies were pooled using meta-analysis when possible. DerSimonian and Laird random effects models were used when heterogeneity was detected; otherwise, a fixed effects Mantel-Haenszel method was applied. It is worth noting that a random effects meta-analysis awards more weight to smaller studies than a fixed effects meta-analysis, and the weights for each study tend to equality as the between-trial variance increases.

### Assessment of Heterogeneity

Data that could not be presented in forest plots were tabulated. Heterogeneity was assessed by visually inspecting the forest plots to detect overlapping confidence intervals, applying the chi-squared test with a *p*-value of 0.10 used to indicate statistical significance, and implementing the *I* ^2^ test statistic, with a value of 50% indicating a moderate level of heterogeneity. Of course, such assessments of heterogeneity are influenced by the number of included studies and should be interpreted with caution.

Heterogeneity was substantive and common in all the analyses, and we sought explanations through a variety of pre-specified subgroup analyses. Subgroups included net type, type and concentration of insecticide, and whether the net was washed or not. We carried out sensitivity analyses by examining the effects when analyses were restricted to hut trials that had a low risk of bias (i.e., ITNs randomly allocated to huts, ITNs rotated, sleepers rotated). Reporting biases were explored using funnel plots. We calculated the confidence intervals for the *I* ^2^ statistic using the method described in [27].

## Results

### Search Results

**Figure 1:**
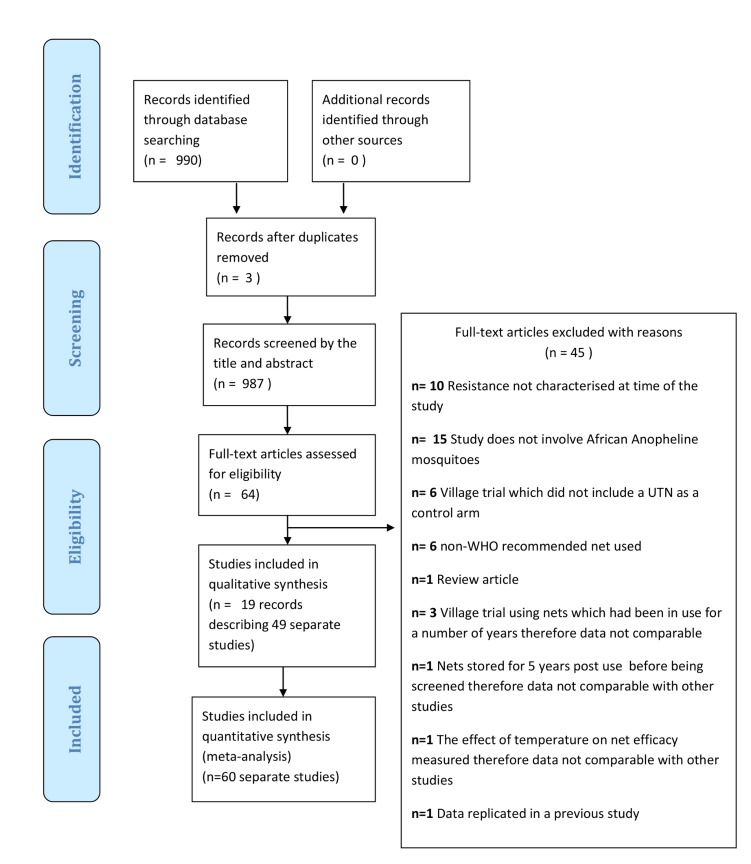
Flow diagram of the study selection process.

Figure 1 displays the review profile. Database searches recovered 1,107 records, from which three duplicates were removed. Searching other sources did not yield any potentially relevant records. After screening the 1,104 records, 914 records were excluded. Of the remaining 73 records, 55 records were excluded (see Figure 1 for exclusion reasons). The remaining 25 records [4],[6],[9],[28]–[49] described 60 separate studies (a study is defined as a comparison that has a distinct control UTN arm). Results of 53 of the 60 studies were combined in a meta-analysis; the results of five studies are described in Tables 5 and 6; and two studies did not report useable data.

**Table 5:** Results from cone tests comparing LLIN or CTN versus UTN for time to 50% and 95% knock-down.

Study	Intervention (All versus UTN)	Net Washed	Mosquito Species (Strain)	Resistance Status	ITN			UTN		
					KDT_50_ (min)	KDT_95_ (min)	Total Number of Mosquitoes Tested	KDT_50_ (min)	KDT_95_ (min)	Total Number of Mosquitoes Tested
Hodjati 1999 (KWA 1 d) [44]	CTN permethrin 500 mg/m^2^	No	*An. gambiae* s.s. (KWA)	Low	11.7	—	110	NA	NA	33
Hodjati 1999 (KWA 10 d fed) [44]	CTN permethrin 500 mg/m^2^	No	*An. gambiae* s.s. (KWA)	Low	10.4	—	110	NA	NA	33
Mahama 2007 (Kisumu) [46]	LLIN PermaNet 2.0	No	*An. gambiae* s.s. (Kisumu)	Low	5	10	50	NA	NA	50
Fane 2012 [47]	CTN alpha-cypermethrin 40 mg/m^2^	No	*An. gambiae* s.s. (Kisumu)	Low	<3.0	26	400	NA	NA	400
Hodjati 1999 (RSP 1 d) [44]	CTN permethrin 500 mg/m^2^	No	*An. gambiae* s.s. (RSP)	High	23.2	—	132	NA	NA	33
Hodjati 1999 (RSP 10 d) [44]	CTN permethrin 500 mg/m^2^	No	*An. gambiae* s.s. (RSP)	High	16.6	—	77	NA	NA	33
Mahama 2007 (VKPR) [46]	LLIN PermaNet 2.0	No	*An. gambiae* s.s. (VKPR)	High	16.4	32.7	100	NA	NA	100

KDT_50_, time to knock-down of 50% of the mosquitoes; KDT_95_, time to knock-down of 95% of the mosquitoes; NA, not applicable.

**Table 6:** Results from tunnel tests comparing CTN versus UTN for mosquito mortality, blood feeding, and not passed though net.

Study	Intervention (All versus UTN)	Net Washed	Mosquito Species (Strain)	Resistance Status	Mortality (Percent)		Blood Feeding (Percent)		Not Passed though Net (Percent)	
					ITN	UTN	ITN	UTN	ITN	UTN
Oxborough 2009a [40]	CTN deltamethrin 25 mg/m^2^ (on polyester nets)	No	*An. gambiae* s.s. (Kisumu)	Low	87	Not stated	28	Not stated	53	Not stated
Oxborough 2009b [40]	CTN deltamethrin 25 mg/m^2^ (on polyethylene nets)	No	*An. gambiae* s.s. (Kisumu)	Low	97	Not stated	11	Not stated	67	Not stated
Oxborough 2009c [40]	CTN deltamethrin 25 mg/m^2^ (on cotton nets)	No	*An. gambiae* s.s. (Kisumu)	Low	85	Not stated	28	Not stated	58	Not stated
Oxborough 2009d [40]	CTN deltamethrin 25 mg/m^2^ (on nylon nets)	No	*An. gambiae* s.s. (Kisumu)	Low	63	Not stated	50	Not stated	46	Not stated

The updated MEDLINE search (May–December 2013) recovered 291 records, of which two records were assessed for eligibility. They were subsequently excluded for not meeting the study design inclusion criteria and for not characterising resistance in the mosquito populations at the time of the study.

### Characteristics of Included Studies

The 60 included studies included cone tests (*n* = 25), tunnel tests (*n* = 11), and experimental hut trials (*n* = 24).

#### Cone tests

The 25 included cone test studies made 60 comparisons. Characteristics for each comparison are given in Table 7. UTNs were compared against unwashed and washed CTNs and LLINs.

**Table 7:** Study characteristics of the included cone tests.

Study	Mosquito Species (Strain/Origin)	Intervention (All versus UTN)	ITN Washed	Resistance Status	Bioassay Percent Mortality (Insecticide)	*kdr* Frequency (Mutation)	Metabolic Resistance	Measured Outcomes			
								MM	KD	KDT_50_	KDT_95_
Darriet 1998 (Kisumu)a [32]	*An. gambiae* s.s. (Kisumu, lab strain)	CTN deltamethrin 25 mg/m^2^	No	Low	100% (permethrin 0.25%), 100% (deltamethrin 0.25%)	Not stated	Not stated	Y	Y	N	N
Darriet 1998 (Kisumu)b [32]	*An. gambiae* s.s. (Kisumu, lab strain)	CTN permethrin 500 mg/m^2^, 225 holes	No	Low	100% (permethrin 0.25%), 100% (deltamethrin 0.25%)	Not stated	Not stated	Y	Y	N	N
Darriet 1998 (YFO)a [32]	*An. gambiae* s.s. (Yaokoffikro, Côte d'Ivoire, wild population)	CTN deltamethrin 25 mg/m^2^, 225 holes	No	High	67.0% (deltamethrin 0.025%)	>80% (L1014F)	Not stated	Y	Y	N	N
Darriet 1998 (YFO)b [32]	*An. gambiae* s.s. (Yaokoffikro, Côte d'Ivoire, wild population)	CTN permethrin 500 mg/m^2^, 225 holes	No	High	15.9% (permethrin 0.25%)	>80% (L1014F)	Not stated	Y	Y	N	N
Etang 2004 (Kisumu) [43]	*An. gambiae* s.s. (Kisumu, lab strain)	CTN permethrin 500 mg/m^2^	No	Low	Not stated	Not stated	Not stated	Y	Y	N	N
Etang 2004 (OC-Lab) [43]	*An. gambiae* (lab strain)	CTN permethrin 500 mg/m^2^	No	Unclear	Not stated	Not stated	Elevated P450 activity	Y	Y	N	N
Fane 2012 [47]	*An. gambiae* s.s. (Kisumu, lab strain)	CTN alpha-cypermethrin 40 mg/m^2^	No	Low	Not stated	Not stated	Not stated	Y	N	Y	Y
Gimnig 2005 (Kisumu)a [45]	*An. gambiae* s.s. (Kisumu, lab strain)	LLIN Olyset	No	Low	Not stated	Not stated	Not stated	Y	Y	N	N
Gimnig 2005 (Kisumu)b [45]	*An. gambiae* s.s. (Kisumu, lab strain)	CTN K-O Tab 1-2-3 deltamethrin 25 mg/m^2^	No	Low	Not stated	Not stated	Not stated	Y	Y	N	N
Hodjati 1999 (KWA 1 d) [44]	*An. gambiae* s.s. (KWA, lab strain)	CTN permethrin 500 mg/m^2^	No	Low	Not stated	Not stated	Not stated	Y	N	Y	N
Hodjati 1999 (KWA 10 d) [44]	*An. gambiae* s.s. (KWA, lab strain)	CTN permethrin 500 mg/m^2^	No	Low	Not stated	Not stated	Not stated	Y	N	Y	N
Hodjati 1999 (KWA 10 d fed) [44]	*An. gambiae* s.s. (KWA, lab strain)	CTN permethrin 500 mg/m^2^	No	Low	Not stated	Not stated	Not stated	Y	N	Y	N
Hodjati 1999 (RSP 1 d) [44]	*An. gambiae* s.s. (RSP, lab strain)	CTN permethrin 500 mg/m^2^	No	High	Not stated	Not stated	Not stated	Y	N	Y	N
Hodjati 1999 (RSP 10 d) [44]	*An. gambiae* s.s. (RSP, lab strain)	CTN permethrin 500 mg/m^2^	No	High	Not stated	Not stated	Not stated	Y	N	Y	N
Hodjati 1999 (RSP 10 d fed) [43]	*An. gambiae* s.s. (RSP, lab strain)	CTN permethrin 500 mg/m^2^	No	High	Not stated	Not stated	Not stated	Y	N	Y	N
Mahama 2007 (Kisumu) [46]	*An. gambiae* s.s. (Kisumu, lab strain)	LLIN PermaNet 2.0	No	Low	Not stated	Not stated	Not stated	Y	N	Y	Y
Mahama 2007 (VKPR) [46]	*An. gambiae* s.s. (VKPR, lab strain)	LLIN PermaNet 2.0	No	High	Not stated	Not stated	Not stated	Y	N	Y	Y
Malima 2009 (cone) [37]	*An. gambiae* s.s. (Muheza, Tanzania, wild population)	CTN deltamethrin 25 mg/m^2^	No	Low	100% (permethrin 0.75%)	Not stated	Not stated	Y	Y	N	N
Koudou 2011 (Kisumu)a [42]	*An. gambiae* s.s. (Kisumu, lab strain)	LLIN PermaNet 3.0	No	Low	Not stated	Not stated	Not stated	Y	Y	N	N
Koudou 2011 (Kisumu)b [42]	*An. gambiae* s.s. (Kisumu, lab strain)	LLIN PermaNet 3.0	Yes	Low	Not stated	Not stated	Not stated	Y	Y	N	N
Koudou 2011 (Kisumu)c [42]	*An. gambiae* s.s. (Kisumu, lab strain)	LLIN PermaNet 2.0	No	Low	Not stated	Not stated	Not stated	Y	Y	N	N
Koudou 2011 (Kisumu)d [42]	*An. gambiae* s.s. (Kisumu, lab strain)	LLIN PermaNet 2.0	Yes	Low	Not stated	Not stated	Not stated	Y	Y	N	N
Koudou 2011 (Kisumu)e [42]	*An. gambiae* s.s. (Kisumu, lab strain)	CTN deltamethrin 25 mg/m^2^	Yes	Low	Not stated	Not stated	Not stated	Y	Y	N	N
Koudou 2011 (YFO)a [42]	*An. gambiae* s.s. (Yaokoffikro, Côte d'Ivoire, wild population)	LLIN PermaNet 3.0	No	High	10.6% (deltamethrin 0.05%)	>80% (L1014F)	Not stated	Y	Y	N	N
Koudou 2011 (YFO)b [42]	*An. gambiae* s.s. (Yaokoffikro, Côte d'Ivoire, wild population)	LLIN PermaNet 3.0	Yes	High	10.6% (deltamethrin 0.05%)	>80% (L1014F)	Not stated	Y	Y	N	N
Koudou 2011 (YFO)c [42]	*An. gambiae* s.s. (Yaokoffikro, Côte d'Ivoire, wild population)	LLIN PermaNet 2.0	No	High	10.6% (deltamethrin 0.05%)	>80% (L1014F)	Not stated	Y	Y	N	N
Koudou 2011 (YFO)d [42]	*An. gambiae* s.s. (Yaokoffikro, Côte d'Ivoire, wild population)	LLIN PermaNet 2.0	Yes	High	10.6% (deltamethrin 0.05%)	>80% (L1014F)	Not stated	Y	Y	N	N
Koudou 2011 (YFO)e [42]	*An. gambiae* s.s. (Yaokoffikro, Côte d'Ivoire, wild population)	CTN deltamethrin 25 mg/m^2^	Yes	High	10.6% (deltamethrin 0.05%)	>80% (L1014F)	Not stated	Y	Y	N	N
Malima 2008 (cone)a [36]	*An. gambiae* s.s. (Kisumu, lab strain)	LLIN Olyset	No	Low	Not stated	Not stated	Not stated	Y	Y	N	N
Malima 2008 (cone)b [36]	*An. gambiae* s.s. (Kisumu, lab strain)	CTN alpha-cypermethrin 20 mg/m^2^	No	Low	Not stated	Not stated	Not stated	Y	Y	N	N
Okia 2013 (Kisumu)a [4]	*An. gambiae* s.s. (Kisumu, lab strain)	LLIN Olyset	No	Low	100% (permethrin 0.75%), 100% (deltamethrin 0.05%)	Not stated	Not stated	Y	N	N	N
Okia 2013 (Kisumu)b [4]	*An. gambiae* s.s. (Kisumu, lab strain)	LLIN Interceptor	No	Low	100% (permethrin 0.75%), 100% (deltamethrin 0.05%)	Not stated	Not stated	Y	N	N	N
Okia 2013 (Kisumu)c [4]	*An. gambiae* s.s. (Kisumu, lab strain)	LLIN Netprotect	No	Low	100% (permethrin 0.75%), 100% (deltamethrin 0.05%)	Not stated	Not stated	Y	N	N	N
Okia 2013 (Kisumu)d [4]	*An. gambiae* s.s. (Kisumu, lab strain)	LLIN PermaNet 2.0	No	Low	100% (permethrin 0.75%), 100% (deltamethrin 0.05%)	Not stated	Not stated	Y	N	N	N
Okia 2013 (Kisumu)e [4]	*An. gambiae* s.s. (Kisumu, lab strain)	LLIN PermaNet 3.0	No	Low	100% (permethrin 0.75%), 100% (deltamethrin 0.05%)	Not stated	Not stated	Y	N	N	N
Okia 2013 (Kanugu)a [4]	*An. gambiae* s.s. (Kanugu, Uganda, wild population)	LLIN Olyset	No	Moderate	68% (permethrin 0.75%), 97% (deltamethrin 0.05%)	36.7% (L1014S)	Not stated	Y	N	N	N
Okia 2013 (Kanugu)b [4]	*An. gambiae* s.s. (Kanugu, Uganda, wild population)	LLIN Interceptor	No	Moderate	68% (permethrin 0.75%), 97% (deltamethrin 0.05%)	36.7% (L1014S)	Not stated	Y	N	N	N
Okia 2013 (Kanugu)c [4]	*An. gambiae* s.s. (Kanugu, Uganda, wild population)	LLIN Netprotect	No	Moderate	68% (permethrin 0.75%), 97% (deltamethrin 0.05%)	36.7% (L1014S)	Not stated	Y	N	N	N
Okia 2013 (Kanugu)d [4]	*An. gambiae* s.s. (Kanugu, Uganda, wild population)	LLIN PermaNet 2.0	No	Moderate	68% (permethrin 0.75%), 97% (deltamethrin 0.05%)	36.7% (L1014S)	Not stated	Y	N	N	N
Okia 2013 (Kanugu)e [4]	*An. gambiae* s.s. (Kanugu, Uganda, wild population)	LLIN PermaNet 3.0	No	Moderate	68% (permethrin 0.75%), 97% (deltamethrin 0.05%)	36.7% (L1014S)	Not stated	Y	N	N	N
Okia 2013 (Lira)a [4]	*An. gambiae* s.s. (Lira, Uganda, wild population)	LLIN Olyset	No	Moderate	60% (permethrin 0.75%), 71% (deltamethrin 0.05%)	33.5% (L1014S)	Not stated	Y	N	N	N
Okia 2013 (Lira)b [4]	*An. gambiae* s.s. (Lira, Uganda, wild population)	LLIN Interceptor	No	Moderate	60% (permethrin 0.75%), 71% (deltamethrin 0.05%)	33.5% (L1014S)	Not stated	Y	N	N	N
Okia 2013 (Lira)c [4]	*An. gambiae* s.s. (Lira, Uganda, wild population)	LLIN Netprotect	No	Moderate	60% (permethrin 0.75%), 71% (deltamethrin 0.05%)	33.5% (L1014S)	Not stated	Y	N	N	N
Okia 2013 (Lira)d [4]	*An. gambiae* s.s. (Lira, Uganda, wild population)	LLIN PermaNet 2.0	No	Moderate	60% (permethrin 0.75%), 71% (deltamethrin 0.05%)	33.5% (L1014S)	Not stated	Y	N	N	N
Okia 2013 (Lira)e [4]	*An. gambiae* s.s. (Lira, Uganda, wild population)	LLIN PermaNet 3.0	No	Moderate	60% (permethrin 0.75%), 71% (deltamethrin 0.05%)	33.5% (L1014S)	Not stated	Y	N	N	N
Okia 2013 (Tororo)a [4]	*An. gambiae* s.s. (Tororo, Uganda, wild population)	LLIN Olyset	No	Moderate	53% (permethrin 0.75%), 66% (deltamethrin 0.05%)	35.4% (L1014S)	Not stated	Y	N	N	N
Okia 2013 (Tororo)b [4]	*An. gambiae* s.s. (Tororo, Uganda, wild population)	LLIN Interceptor	No	Moderate	53% (permethrin 0.75%), 66% (deltamethrin 0.05%)	35.4% (L1014S)	Not stated	Y	N	N	N
Okia 2013 (Tororo)c [4]	*An. gambiae* s.s. (Tororo, Uganda, wild population)	LLIN Netprotect	No	Moderate	53% (permethrin 0.75%), 66% (deltamethrin 0.05%)	35.4% (L1014S)	Not stated	Y	N	N	N
Okia 2013 (Tororo)d [4]	*An. gambiae* s.s. (Tororo, Uganda, wild population)	LLIN PermaNet 2.0	No	Moderate	53% (permethrin 0.75%), 66% (deltamethrin 0.05%)	35.4% (L1014S)	Not stated	Y	N	N	N
Okia 2013 (Tororo)e [4]	*An. gambiae* s.s. (Tororo, Uganda, wild population)	LLIN PermaNet 3.0	No	Moderate	53% (permethrin 0.75%), 66% (deltamethrin 0.05%)	35.4% (L1014S)	Not stated	Y	N	N	N
Okia 2013 (Wakiso)a [4]	*An. gambiae* s.s. (Wakiso, Uganda, wild population)	LLIN Olyset	No	Moderate	90% (permethrin 0.75%), 94% (deltamethrin 0.05%)	36.6% (L1014S)	Not stated	Y	N	N	N
Okia 2013 (Wakiso)b [4]	*An. gambiae* s.s. (Wakiso, Uganda, wild population)	LLIN Interceptor	No	Moderate	90% (permethrin 0.75%), 94% (deltamethrin 0.05%)	36.6% (L1014S)	Not stated	Y	N	N	N
Okia 2013 (Wakiso)c [4]	*An. gambiae* s.s. (Wakiso, Uganda, wild population)	LLIN Netprotect	No	Moderate	90% (permethrin 0.75%), 94% (deltamethrin 0.05%)	36.6% (L1014S)	Not stated	Y	N	N	N
Okia 2013 (Wakiso)d [4]	*An. gambiae* s.s. (Wakiso, Uganda, wild population)	LLIN PermaNet 2.0	No	Moderate	90% (permethrin 0.75%), 94% (deltamethrin 0.05%)	36.6% (L1014S)	Not stated	Y	N	N	N
Okia 2013 (Wakiso)e [4]	*An. gambiae* s.s. (Wakiso, Uganda, wild population)	LLIN PermaNet 3.0	No	Moderate	90% (permethrin 0.75%), 94% (deltamethrin 0.05%)	36.6% (L1014S)	Not stated	Y	N	N	N
Okumu 2012a [6]	*An. arabiensis* (colony established from wild population)	LLIN Icon Life	No	Low	100% (DDT 4%), >90% (pyrethroids)	Not stated	Not stated	Y	N	N	N
Okumu 2012b [6]	*An. arabiensis* (colony established from wild population)	LLIN Olyset	No	Low	100% (DDT 4%), >90% (pyrethroids)	Not stated	Not stated	Y	N	N	N
Okumu 2012c [6]	*An. arabiensis* (colony established from wild population)	LLIN PermaNet 2.0	No	Low	100% (DDT 4%), >90% (pyrethroids)	Not stated	Not stated	Y	N	N	N
Winkler 2012a [48]	*An. gambiae* s.s. (Kisumu, lab strain)	CTN Icon Maxx lambda-cyhalothrin (polyethylene net)	No	Low	Not stated	Not stated	Not stated	Y	N	N	N
Winkler 2012b [48]	*An. gambiae* s.s. (Kisumu, lab strain)	CTN Icon Maxx lambda-cyhalothrin (polyester net)	No	Low	Not stated	Not stated	Not stated	Y	N	N	N

KD, percent knock-down at 60 min; KDT_50_, time to knock-down of 50% of the mosquitoes; KDT_95_, time to knock-down of 95% of the mosquitoes; MM, mosquito mortality; OC-Lab, OCEAC Laboratory strain; YFO, Yaokoffikro.

Fifty-seven comparisons used *An. gambiae* s.s. mosquitoes, whilst three were of *An. arabiensis*. Overall, 29 comparisons used laboratory-reared mosquito strains (Kisumu, VKPR, OC-Lab, KWA, and RSP strains), and 28 comparisons used wild field-caught mosquitoes from Yaokoffikro (Côte d'Ivoire), Muheza (Tanzania), and localities in Uganda. Three comparisons used recently colonised *An. arabiensis* mosquitoes that were originally collected from the Ulanga District of Tanzania.

Based on the reported WHO bioassay percent mortalities and *kdr* frequencies, 28 comparisons were carried out with mosquitoes with low resistance, 20 comparisons with moderately resistant mosquitoes, and 11 comparisons with highly resistant mosquitoes; resistance was unclear for one comparison. Only one comparison measured metabolic resistance.

For the risk of bias assessment, all comparisons reported comparability of ITN and UTN mosquito groups, but it was unclear in all studies whether observers were blinded (Table S6). No comparison reported incomplete outcome data. Fifteen comparisons reported raw data for ITN and UTN groups, the remaining 45 did not.

#### Tunnel tests

The 11 included tunnel test studies made 20 comparisons. UTNs were compared against unwashed CTNs and LLINs. Characteristics for each comparison are given in Table 8. All comparisons used *An. gambiae* mosquitoes (the number of mosquitoes used varied from 200 to 592). Three comparisons used wild field-caught mosquitoes from Yaokoffikro (Côte d'Ivoire) and Muheza (Tanzania) in their assessment, whilst 17 comparisons used laboratory-reared mosquito strains (Kisumu, VKPR, Kisumu/VKPR hybrids, Tola, and Kou strains). Based on the reported WHO bioassay percent mortalities and *kdr* frequencies, 12 comparisons were carried out with mosquitoes with low resistance, six comparisons used highly resistant mosquitoes, and resistance was moderate for two comparisons. No comparison measured metabolic resistance.

**Table 8:** Study characteristics of the included tunnel tests.

Study	Mosquito Species (Strain/Origin)	Intervention (All versus UTN)	Net Washed	Resistance Status	Resistance Testing			Measured Outcomes		
					Bioassay Percent Mortality (Insecticide)	*kdr* Frequency (Mutation)	Metabolic Resistance	MM	BF	NPT
Chandre 2000 (L1 Kisumu) [29]	*An. gambiae* s.s. (Kisumu, lab strain)	CTN permethrin 250 mg/m^2^	No	Low	98% (permethrin 0.25%)	Not stated	Not stated	Y	Y	Y
Chandre 2000 (L1 Kou) [29]	*An. gambiae* (Kou, lab strain)	CTN permethrin 250 mg/m^2^	No	High	0% (permethrin 0.25%)	100% (L1014F)	Not stated	Y	Y	Y
Chandre 2000 (L1 Tola) [29]	*An. gambiae* s.s. (Tola, lab strain)	CTN permethrin 250 mg/m^2^	No	High	Not stated	100% (L1014F)	Not stated	Y	Y	Y
Chandre 2000 (L2 Kisumu) [29]	*An. gambiae* s.s. (Kisumu, lab strain)	CTN permethrin 500 mg/m^2^	No	Low	98% (permethrin 0.25%)	Not stated	Not stated	Y	Y	N
Chandre 2000 (L2 YFO)a [29]	*An. gambiae* s.s. (Yaokoffikro, Côte d'Ivoire, wild population)	CTN permethrin 250 mg/m^2^	No	High	Not stated	94.4% (L1014F)	Not stated	Y	Y	N
Chandre 2000 (L2 YFO)b [29]	*An. gambiae* s.s. (Yaokoffikro, Côte d'Ivoire, wild population)	CTN permethrin 500 mg/m^2^	No	High	Not stated	94.4% (L1014F)	Not stated	Y	Y	N
Corbel 2004 (Kisumu/VKPR hybrid)a [30]	*An. gambiae* (Kisumu/VKPR hybrid, lab strain)	CTN permethrin 250 mg/m^2^	No	Moderate	Not stated	RS (frequency not stated)	Not stated	Y	Y	N
Corbel 2004 (Kisumu/VKPR hybrid)b [30]	*An. gambiae* (Kisumu/VKPR hybrid, lab strain)	CTN permethrin 500 mg/m^2^	No	Moderate	Not stated	RS (frequency not stated)	Not stated	Y	Y	N
Corbel 2004 (Kisumu)a [30]	*An. gambiae* s.s. (Kisumu, lab strain)	CTN permethrin 250 mg/m^2^	No	Low	Not stated	Not stated	Not stated	Y	Y	N
Corbel 2004 (Kisumu)b [30]	*An. gambiae* s.s. (Kisumu, lab strain)	CTN permethrin 500 mg/m^2^	No	Low	Not stated	Not stated	Not stated	Y	Y	N
Corbel 2004 (VKPR)a [30]	*An. gambiae* s.s. (VKPR, lab strain)	CTN permethrin 250 mg/m^2^	No	High	Not stated	RR (frequency not stated)	Not stated	Y	Y	N
Corbel 2004 (VKPR)b [30]	*An. gambiae* s.s. (VKPR, lab strain)	CTN permethrin 500 mg/m^2^	No	High	Permethrin resistant	RR (frequency not stated)	Not stated	Y	Y	N
Malima 2008a [36]	*An. gambiae* s.s. (Kisumu, lab strain)	CTN alpha-cypermethrin 20 mg/m^2^	No	Low	100% (deltamethrin 0.05%), 100% (permethrin 0.75%)	absent	Not stated	Y	Y	Y
Malima 2008b [36]	*An. gambiae* s.s. (Kisumu, lab strain)	LLIN Olyset	No	Low	100% (permethrin 0.75%)	Not stated	Not stated	Y	Y	Y
Malima 2009 (tunnel) [37]	*An. gambiae* s.s. (Muheza, Tanzania, wild population)	CTN deltamethrin 25 mg/m^2^	No	Low	100% (permethrin 0.75%)	Not stated	Not stated	Y	Y	Y
Oxborough 2009a [40]	*An. gambiae* s.s. (Kisumu, lab strain)	CTN deltamethrin 25 mg/m^2^ (on polyester nets)	No	Low	Not stated	Not stated	Not stated	Y	Y	Y
Oxborough 2009b [40]	*An. gambiae* s.s. (Kisumu, lab strain)	CTN deltamethrin 25 mg/m^2^ (on polyethylene nets)	No	Low	Not stated	Not stated	Not stated	Y	Y	Y
Oxborough 2009c [40]	*An. gambiae* s.s. (Kisumu, lab strain)	CTN deltamethrin 25 mg/m^2^ (on cotton nets)	No	Low	Not stated	Not stated	Not stated	Y	Y	Y
Oxborough 2009d [40]	*An. gambiae* s.s. (Kisumu, lab strain)	CTN deltamethrin 25 mg/m^2^ (on nylon nets)	No	Low	Not stated	Not stated	Not stated	Y	Y	Y

BF, blood fed; MM, mosquito mortality; NPT, not passed through net; RR, homozygous for the *kdr* allele; RS, heterozygous for the *kdr* allele.

For the risk of bias assessment, 16 comparisons reported comparability of ITN and UTN mosquito groups, whilst comparability was unclear in four comparisons (Table S7). It was unclear in all studies whether observers were blinded. No comparison reported incomplete outcome data. Sixteen comparisons reported raw data for ITN and UTN groups, the remaining four did not.

#### Experimental hut field trials

The 24 included hut studies made 56 comparisons (Table 9). 20 comparisons used field sites in Côte D'Ivoire, 14 in Tanzania, 11 in Benin, six in Burkina Faso, and five in Cameroon. Most comparisons (41 of 56) were of *An. gambiae* mosquitoes, 12 were of *An. arabiensis*, and three were of *An. funestus*. Two comparisons used laboratory-reared strains (Kisumu). Based on the reported WHO bioassay percent mortalities and *kdr* frequencies, 26 comparisons were carried out with mosquitoes with low resistance, 21 comparisons used highly resistant mosquitoes, and resistance was moderate for nine comparisons. Two comparisons measured metabolic resistance.

**Table 9:** Study characteristics of the included experimental hut trials.

Study	Study Location	Study Start Date	Duration (Nights)	Mosquito Species (Strain/Origin)	Intervention (All versus UTN)	Net Washed	Resistance Status	Resistance Testing			Measured Outcomes			
								WHO Bioassay Percent Mortality (Insecticide)	*kdr* Frequency (L1014F Mutation)	Metabolic Resistance	D	BF	IE	MM
Asidi 2005a [28]	Yaokoffikro field station, Côte d'Ivoire	15 August 2002	33	*An. gambiae* s.s.	CTN lambda-cyalothrin 18 mg/m^2^	No	High	NS	>90%^a^	NS	Y	Y	Y	Y
Asidi 2005b [28]	Yaokoffikro field station, Côte d'Ivoire	15 August 2002	33	*An. gambiae* s.s.	CTN lambda-cyalothrin 18 mg/m^2^	Yes	High	NS	>90%^a^	NS	Y	Y	Y	Y
Chandre 2000 (Kisumu)a [29]	Yaokoffikro field station, Côte d'Ivoire	NS	NS	*An. gambiae* s.s. (Kisumu, lab strain)	CTN deltamethrin 25 mg/m^2^	No	Low	98.6% (permethrin 0.25%)	NS	NS	Y	Y	N	Y
Chandre 2000 (Kisumu)b [29]	Yaokoffikro field station, Côte d'Ivoire	NS	NS	*An. gambiae* s.s. (Kisumu, lab strain)	CTN permethrin 500 mg/m^2^	No	Low	98.6% (permethrin 0.25%)	NS	NS	Y	Y	N	Y
Chandre 2000 (YFO)a [29]	Yaokoffikro field station, Côte d'Ivoire	NS	NS	*An. gambiae* s.s. (Yaokoffikro, wild population)	CTN deltamethrin 25 mg/m^2^	No	High	NS	94.40%	NS	Y	Y	N	Y
Chandre 2000 (YFO)b [29]	Yaokoffikro field station, Côte d'Ivoire	NS	NS	*An. gambiae* s.s. (Yaokoffikro, wild population)	CTN permethrin 500 mg/m^2^	No	High	NS	94.40%	NS	Y	Y	N	Y
Corbel 2004a [30]	CREC field station, Cotonou, Benin	NS	NS	*An. gambiae* s.s. (M form)	CTN permethrin 500 mg/m^2^	No	Moderate	NS	78.80%	NS	Y	Y	Y	Y
Corbel 2004b [30]	CREC field station, Cotonou, Benin	NS	NS	*An. gambiae* s.s. (M form)	CTN permethrin 250 mg/m^2^	No	Moderate	NS	63.40%	NS	Y	Y	Y	Y
Corbel 2010 (Benin)a [31]	Malanville, Benin	NS	NS	*An. gambiae* s.s. (S form)	LLIN PermaNet 2.0	No	Low	85% (deltamethrin 0.05%)	16%	NS	Y	Y	Y	Y
Corbel 2010 (Benin)b [31]	Malanville, Benin	NS	NS	*An. gambiae* s.s. (S form)	LLIN PermaNet 2.0	Yes	Low	85% (deltamethrin 0.05%)	16%	NS	Y	Y	Y	Y
Corbel 2010 (Benin)c [31]	Malanville, Benin	NS	NS	*An. gambiae* s.s. (S form)	LLIN PermaNet 3.0	No	low	85% (deltamethrin 0.05%)	16%	NS	Y	Y	Y	Y
Corbel 2010 (Benin)d [31]	Malanville, Benin	NS	NS	*An. gambiae* s.s. (S form)	LLIN PermaNet 3.0	Yes	Low	85% (deltamethrin 0.05%)	16%	NS	Y	Y	Y	Y
Corbel 2010 (Benin)e [31]	Malanville, Benin	NS	NS	*An. gambiae* s.s. (S form)	CTN deltamethrin 25 mg/m^2^	Yes	Low	85% (deltamethrin 0.05%)	16%	NS	Y	Y	Y	Y
Corbel 2010 (BFaso)a [31]	Valleé du Kou, Burkina Faso	NS	NS	*An. gambiae* s.s. (15% M form/85% S form)	LLIN PermaNet 2.0	No	High	23% (deltamethrin 0.05%)	>80%	NS	Y	Y	Y	Y
Corbel 2010 (BFaso)b [31]	Valleé du Kou, Burkina Faso	NS	NS	*An. gambiae* s.s. (15% M form/85% S form)	LLIN PermaNet 2.0	Yes	High	23% (deltamethrin 0.05%)	>80%	NS	Y	Y	Y	Y
Corbel 2010 (BFaso)c [31]	Valleé du Kou, Burkina Faso	NS	NS	*An. gambiae* s.s. (15% M form/85% S form)	LLIN PermaNet 3.0	No	High	23% (deltamethrin 0.05%)	>80%	NS	Y	Y	Y	Y
Corbel 2010 (BFaso)d [31]	Valleé du Kou, Burkina Faso	NS	NS	*An. gambiae* s.s. (15% M form/85% S form)	LLIN PermaNet 3.0	Yes	High	23% (deltamethrin 0.05%)	>80%	NS	Y	Y	Y	Y
Corbel 2010 (BFaso)e [31]	Valleé du Kou, Burkina Faso	NS	NS	*A An. gambiae* s.s. (15% M form/85% S form)	CTN deltamethrin 25 mg/m^2^	Yes	High	23% (deltamethrin 0.05%)	>80%	NS	Y	Y	Y	Y
Corbel 2010 (Cameroon)a [30]	Pitoa, Cameroon	NS	NS	*An. arabiensis* (95%), *An. gambiae* s.s. (5%) (S form)	LLIN PermaNet 2.0	No	Moderate	70% (deltamethrin 0.05%)	<5%	NS	Y	Y	Y	Y
Corbel 2010 (Cameroon)b [31]	Pitoa, Cameroon	NS	NS	*An. arabiensis* (95%), *An. gambiae* s.s. (5%) (S form)	LLIN PermaNet 2.0	Yes	Moderate	70% (deltamethrin 0.05%)	<5%	NS	Y	Y	Y	Y
Corbel 2010 (Cameroon)c [31]	Pitoa, Cameroon	NS	NS	*An. arabiensis* (95%), *An. gambiae* s.s. (5%) (S form)	LLIN PermaNet 3.0	No	Moderate	70% (deltamethrin 0.05%)	<5%	NS	Y	Y	Y	Y
Corbel 2010 (Cameroon)d [31]	Pitoa, Cameroon	NS	NS	*An. arabiensis* (95%), *An. gambiae* s.s. (5%) (S form)	LLIN PermaNet 3.0	Yes	Moderate	70% (deltamethrin 0.05%)	<5%	NS	Y	Y	Y	Y
Corbel 2010 (Cameroon)e [31]	Pitoa, Cameroon	NS	NS	*An. arabiensis* (95%), *An. gambiae* s.s. (5%) (S form)	CTN deltamethrin 25 mg/m^2^	Yes	Moderate	70% (deltamethrin 0.05%)	<5%	NS	Y	Y	Y	Y
Darriet 1998a [32]	Yaokoffikro field station, Côte d'Ivoire	NS	NS	*An. gambiae* s.s.	CTN deltamethrin 25 mg/m^2^	No	Moderate	67.0% (deltamethrin 0.25%)	NS	NS	Y	Y	Y	Y
Darriet 1998b [32]	Yaokoffikro field station, Côte d'Ivoire	NS	NS	*An. gambiae* s.s.	CTN permethrin 500 mg/m^2^	No	High	15.9% (permethrin 0.25%)	NS	NS	Y	Y	Y	Y
Darriet 2000 [33]	M'bé, Bouaké, Côte d'Ivoire	NS	NS	*An. gambiae* s.s.	CTN deltamethrin 25 mg/m^2^	No	Low	96.9% (deltamethrin 0.05%)	NS	NS	Y	Y	Y	Y
Fanello 1999a [35]	Bouaké, Côte d'Ivoire	NS	NS	*An. gambiae* s.s.	CTN alpha-cypermethrin 20 mg/m^2^	No	High	NS	90%	NS	Y	Y	N	Y
Fanello 1999b [35]	Bouaké, Côte d'Ivoire	NS	NS	*An. gambiae* s.s.	CTN etofenprox 200 mg/m^2^	No	High	NS	90%	NS	Y	Y	N	Y
Koudou 2011a [42]	Yaokoffikro field station, Côte d'Ivoire	April 2009	84	*An. gambiae* s.s.	LLIN PermaNet 3.0	No	High	10.6% (deltamethrin 0.05%)	NS	NS	Y	N	Y	Y
Koudou 2011b [42]	Yaokoffikro field station, Côte d'Ivoire	April 2009	84	*An. gambiae* s.s.	LLIN PermaNet 2.0	No	High	10.6% (deltamethrin 0.05%)	NS	NS	Y	N	Y	Y
Koudou 2011c [42]	Yaokoffikro field station, Côte d'Ivoire	April 2009	84	*An. gambiae* s.s.	LLIN PermaNet 3.0	Yes	High	10.6% (deltamethrin 0.05%)	NS	NS	Y	N	Y	Y
Koudou 2011d [42]	Yaokoffikro field station, Côte d'Ivoire	April 2009	84	*An. gambiae* s.s.	LLIN PermaNet 2.0	Yes	High	10.6% (deltamethrin 0.05%)	NS	NS	Y	N	Y	Y
Koudou 2011e [42]	Yaokoffikro field station, Côte d'Ivoire	April 2009	84	*An. gambiae* s.s.	CTN deltamethrin 25 mg/m^2^	Yes	High	10.6% (deltamethrin 0.05%)	NS	NS	Y	N	Y	Y
Malima 2008 (funestus)a [36]	Bouaké, Côte d'Ivoire	NS	NS	*An. funestus*	LLIN Olyset	No	Low	100% (deltamethrin 0.05%)	NS	NS	Y	Y	Y	Y
Malima 2008 (funestus)b [36]	Bouaké, Côte d'Ivoire	NS	NS	*An. funestus*	CTN alpha-cypermethrin 20 mg/m^2^	No	Low	100% (deltamethrin 0.05%), 100% (permethrin 0.75%)	NS	NS	Y	Y	Y	Y
Malima 2008 (gambiae)a [36]	Bouaké, Côte d'Ivoire	NS	NS	*An. gambiae* s.s.	LLIN Olyset	No	Low	100% (deltamethrin 0.05%), 100% (permethrin 0.75%)	NS	NS	Y	Y	Y	Y
Malima 2008 (gambiae)b [36]	Bouaké, Côte d'Ivoire	NS	NS	*An. gambiae* s.s.	CTN alpha-cypermethrin 20 mg/m^2^	No	Low	100% (deltamethrin 0.05%), 100% (permethrin 0.75%)	NS	NS	Y	Y	Y	Y
Malima 2009 (funestus) [36]	Muheza, Tanzania	NS	NS	*An. funestus*	CTN deltamethrin 25 mg/m^2^	No	Low	100% (deltamethrin 0.05%)	NS	NS	Y	Y	Y	N
Malima 2009 (gambiae) [37]	Muheza, Tanzania	NS	NS	*An. gambiae* s.s.	CTN deltamethrin 25 mg/m^2^	No	Low	100% (deltamethrin 0.05%)	NS	NS	Y	Y	Y	N
N'Guessan 2007 (Cotonou) [38]	Ladji, Benin	NS	NS	*An. gambiae* s.s.	CTN lambda-cyalothrin 18 mg/m^2^	No	High	NS	83% lambda-cyalothin (0.05%)	P450 activity	Y	Y	Y	Y
N'Guessan 2007 (M.ville) [38]	Malanville, Benin	NS	NS	*An. gambiae* s.s.	CTN lambda-cyalothrin 18 mg/m^2^	No	Low	NS	6%	P450 activity	Y	Y	Y	Y
Ngufor 2011 (6 holes) [39]	Akron, Benin	NS	NS	*An. gambiae* s.s.	LLIN deltamethrin 55 mg/m^2^, 6 holes in the net	No	High	NS	>80%	NS	Y	Y	Y	Y
Ngufor 2011 (80 holes) [39]	Akron, Benin	NS	NS	*An. gambiae* s.s.	LLIN deltamethrin 55 mg/m^2^, 80 holes in the net	No	High	NS	>80%	NS	Y	Y	Y	Y
Okumu 2013 (dry season)a [9]	Ulanga District, Tanzania	NS	NS	*An. arabiensis*	LLIN Olyset	No	Low	100% (DDT), 95.5% (deltamethrin), 95.2% (permethrin), 90.2% (lambda-cyhalothrin)	NS	NS	Y	Y	Y	Y
Okumu 2013 (dry season)b [9]	Ulanga District, Tanzania	NS	NS	*An. arabiensis*	LLIN PermaNet 2.0	No	Low	100% (DDT), 95.5% (deltamethrin), 95.2% (permethrin), 90.2% (lambda-cyhalothrin)	NS	NS	Y	Y	Y	Y
Okumu 2013 (dry season)c [9]	Ulanga District, Tanzania	NS	NS	*An. arabiensis*	LLIN Icon Life	No	Low	100% (DDT), 95.5% (deltamethrin), 95.2% (permethrin), 90.2% (lambda-cyhalothrin)	NS	NS	Y	Y	Y	Y
Okumu 2013 (wet season)a[9]	Ulanga District, Tanzania	NS	NS	*An. arabiensis*	LLIN Olyset	No	Low	100% (DDT), 95.5% (deltamethrin), 95.2% (permethrin), 90.2% (lambda-cyhalothrin)	NS	NS	Y	Y	Y	Y
Okumu 2013 (wet season)b [9]	Ulanga District, Tanzania	NS	NS	*An. arabiensis*	LLIN PermaNet 2.0	No	Low	100% (DDT), 95.5% (deltamethrin), 95.2% (permethrin), 90.2% (lambda-cyhalothrin)	NS	NS	Y	Y	Y	Y
Okumu 2013 (wet season)c [9]	Ulanga District, Tanzania	NS	NS	*An. arabiensis*	LLIN Icon Life	No	Low	100% (DDT), 95.5% (deltamethrin), 95.2% (permethrin), 90.2% (lambda-cyhalothrin)	NS	NS	Y	Y	Y	Y
Oxborough 2013 [49]	KCMUC field station, Tanzania	NS	NS	*An. arabiensis*	CTN alpha-cypermethrin 25 mg/m^2^	No	Moderate	58% (lambda-cyhalothrin 0.05%), 76% (permethrin 0.75%), 100% (DDT 4%), 100% (fenitrothrion 1%)	0% (L1014F), 0% (L1014S)^b^	NS	Y	Y	Y	Y
Tungu 2010a [41]	Muheza, Tanzania	NS	NS	*An. gambiae* s.s.	LLIN PermaNet 2.0	No	Low	100% (deltamethrin 0.05%)	NS	NS	Y	Y	Y	Y
Tungu 2010b [41]	Muheza, Tanzania	NS	NS	*An. gambiae* s.s.	LLIN PermaNet 2.0	Yes	Low	100% (deltamethrin 0.05%)	NS	NS	Y	Y	Y	Y
Tungu 2010c [41]	Muheza, Tanzania	NS	NS	*An. gambiae* s.s.	LLIN PermaNet 3.0	No	Low	100% (deltamethrin 0.05%)	NS	NS	Y	Y	Y	Y
Tungu 2010d [41]	Muheza, Tanzania	NS	NS	*An. gambiae* s.s.	LLIN PermaNet 3.0	Yes	Low	100% (deltamethrin 0.05%)	NS	NS	Y	Y	Y	Y
Tungu 2010e [41]	Muheza, Tanzania	NS	NS	*An. gambiae* s.s.	CTN deltamethrin 25 mg/m^2^	Yes	Low	100% (deltamethrin 0.05%)	NS	NS	Y	Y	Y	Y
Djenontin 2010 [34]	Valleé du Kou, Burkina Faso	NS	NS	*An. gambiae* s.s. (M form)	LLIN PermaNet 2.0	No	High	NS	92%	NS	Y	Y	Y	Y

a In mosquitoes from control huts (mosquitoes from the test huts were not screened).

b Oxborough et al. [49] was the only study that tested for L104F and for L104S, but found no mutations for either.

BF, blood fed; BFaso, Burkina Faso; CREC, Entomological Research Centre of Cotonou; D, deterrence; IE, induced exophily; KCMUC, Kilimanjaro Christian Medical University College; M.ville, Malanville; MM, mosquito mortality; NS, not stated; YFO, Yaokoffikro.

For the risk of bias assessment, no comparisons reported comparability of ITN and UTN mosquito groups or blinded collectors of mosquitoes or the sleepers (Table S8). Forty-eight of the 56 comparisons reported raw data for ITN and UTN groups. It was unclear in 16 comparisons as to whether nets were randomly allocated to huts at the start of the study. Overall, 41 comparisons rotated ITNs, eight did not, and seven did not report rotation. Fifty comparisons rotated sleepers, whilst it was unclear as to whether the remaining comparisons rotated the sleepers between huts.

Table 10 displays the rigor of implementation assessment of each hut trial in terms of particular study design characteristics. Standardisation across studies both in terms of the experimental design and reporting was not consistent. Of the 16 comparisons that compared a washed net, 12 washed the net in accordance with the WHO protocol, one did not wash the net using WHO procedures, and it was unclear whether the remaining three had followed WHO procedures. Seven of the 56 comparisons cleaned the huts before the study, whereas 25 comparisons cleaned the huts after each rotation; the remaining comparisons were unclear regarding when the huts were cleaned. Overall, 38 of the 56 comparisons tested the ITNs before the study, 32 comparisons tested the ITNs on completion of the study, and 22 comparisons tested the nets chemically; the remaining comparisons did not test the nets. Outcomes were not measured on male mosquitoes in 30 of the 56 comparisons, but were measured in the remaining 26 comparisons.

**Table 10:** Assessment of “rigor” for experimental hut trials.

Study	Wash Procedure^a^	Huts Cleaned^b^		ITNs Tested			Male Mosquitoes Excluded from Study	Resistance Testing of Mosquitoes^f^			
		Before Study	After Each Rotation	Before Study^c^	End of Study^d^	Chemically^e^		Bioassays	*kdr*	Number Genotyped Stated^g^	Metabolic Resistance
Asidi 2005a [28]	n/a	Yes	Unclear	No	No	No	Yes	No	Yes	No	No
Asidi 2005b [28]	No	Yes	Unclear	No	No	No	Yes	No	Yes	No	No
Chandre 2000 (Kisumu)a [29]	n/a	Unclear	Unclear	Yes	No	No	Yes	Yes	No	No	No
Chandre 2000 (Kisumu)b [29]	n/a	Unclear	Unclear	Yes	No	No	Yes	Yes	No	No	No
Chandre 2000 (YFO)a [29]	n/a	Unclear	Unclear	Yes	No	No	Yes	No	Yes	No	No
Chandre 2000 (YFO)b [29]	n/a	Unclear	Unclear	Yes	No	No	Yes	No	Yes	No	No
Corbel 2004a [30]	n/a	Unclear	Unclear	No	No	No	Yes	No	Yes	Yes	No
Corbel 2004b [30]	n/a	Unclear	Unclear	No	No	No	Yes	No	Yes	Yes	No
Corbel 2010 (Benin)a [31]	n/a	Unclear	Unclear	Yes	Yes	Yes	No	Yes	Yes	No	No
Corbel 2010 (Benin)b [31]	Yes	Unclear	Unclear	Yes	Yes	Yes	No	Yes	Yes	No	No
Corbel 2010 (Benin)c [31]	n/a	Unclear	Unclear	Yes	Yes	Yes	No	Yes	Yes	No	No
Corbel 2010 (Benin)d [31]	Yes	Unclear	Unclear	Yes	Yes	Yes	No	Yes	Yes	No	No
Corbel 2010 (Benin)e [31]	Yes	Unclear	Unclear	Yes	Yes	Yes	No	Yes	Yes	No	No
Corbel 2010 (BFaso)a [31]	n/a	Unclear	Unclear	Yes	Yes	Yes	No	Yes	Yes	No	No
Corbel 2010 (BFaso)b [31]	Yes	Unclear	Unclear	Yes	Yes	Yes	No	Yes	Yes	No	No
Corbel 2010 (BFaso)c [31]	n/a	Unclear	Unclear	Yes	Yes	Yes	No	Yes	Yes	No	No
Corbel 2010 (BFaso)d [31]	Yes	Unclear	Unclear	Yes	Yes	Yes	No	Yes	Yes	No	No
Corbel 2010 (BFaso)e [31]	Yes	Unclear	Unclear	Yes	Yes	Yes	No	Yes	Yes	No	No
Corbel 2010 (Cameroon)a [31]	n/a	Unclear	Unclear	Yes	Yes	Yes	No	Yes	Yes	No	No
Corbel 2010 (Cameroon)b [31]	Yes	Unclear	Unclear	Yes	Yes	Yes	No	Yes	Yes	No	No
Corbel 2010 (Cameroon)c [31]	n/a	Unclear	Unclear	Yes	Yes	Yes	No	Yes	Yes	No	No
Corbel 2010 (Cameroon)d [31]	Yes	Unclear	Unclear	Yes	Yes	Yes	No	Yes	Yes	No	No
Corbel 2010 (Cameroon)e [31]	Yes	Unclear	Unclear	Yes	Yes	Yes	No	Yes	Yes	No	No
Darriet 1998a [32]	n/a	Unclear	Yes	No	Yes	No	No	Yes	No	n/a	No
Darriet 1998b [32]	n/a	Unclear	Yes	No	Yes	No	No	Yes	No	n/a	No
Darriet 2000 [33]	n/a	Unclear	Unclear	No	No	No	Yes	Yes	No	n/a	No
Fanello 1999a [35]	n/a	Unclear	Unclear	No	No	Yes	No	No	Yes	Yes	No
Fanello 1999b [35]	n/a	Unclear	Unclear	No	No	Yes	No	No	Yes	Yes	No
Koudou 2011a [42]	n/a	Yes	Yes	Yes	Yes	No	Yes	Yes	No	n/a	No
Koudou 2011b [42]	n/a	Yes	Yes	Yes	Yes	No	Yes	Yes	No	n/a	No
Koudou 2011c [42]	Yes	Yes	Yes	Yes	Yes	No	Yes	Yes	No	n/a	No
Koudou 2011d [42]	Yes	Yes	Yes	Yes	Yes	No	Yes	Yes	No	n/a	No
Koudou 2011e [41]	Yes	Yes	Yes	Yes	Yes	No	Yes	Yes	No	n/a	No
Malima 2008 (funestus)a [36]	n/a	Unclear	Yes	Yes	No	No	Yes	Yes	No	n/a	No
Malima 2008 (funestus)b [36]	n/a	Unclear	Yes	Yes	No	No	Yes	Yes	No	n/a	No
Malima 2008 (gambiae)a [36]	n/a	Unclear	Yes	Yes	No	No	Yes	Yes	No	n/a	No
Malima 2008 (gambiae)b [36]	n/a	Unclear	Yes	Yes	No	No	Yes	Yes	No	n/a	No
Malima 2009 (funestus) [37]	n/a	Unclear	Yes	Yes	Yes	No	Yes	Yes	No	n/a	No
Malima 2009 (gambiae) [37]	n/a	Unclear	Yes	Yes	Yes	No	Yes	Yes	No	n/a	No
N'Guessan 2007 (Cotonou) [38]	n/a	Unclear	Unclear	Yes	Yes	No	Yes	Yes	Yes	Yes	Yes
N'Guessan 2007 (M.ville) [38]	n/a	Unclear	Unclear	Yes	Yes	No	Yes	Yes	Yes	Yes	Yes
Ngufor 2011 (6 holes) [39]	n/a	Unclear	Unclear	No	No	No	Yes	No	Yes	Yes	No
Ngufor 2011 (80 holes) [39]	n/a	Unclear	Unclear	No	No	No	Yes	No	Yes	Yes	No
Okumu 2013 (dry season)a [9]	n/a	Unclear	Yes	No	No	No	No	Yes	No	n/a	No
Okumu 2013 (dry season)b [9]	n/a	Unclear	Yes	No	No	No	No	Yes	No	n/a	No
Okumu 2013 (dry season)c [9]	n/a	Unclear	Yes	No	No	No	No	Yes	No	n/a	No
Okumu 2013 (wet season)a [9]	n/a	Unclear	Yes	No	No	No	No	Yes	No	n/a	No
Okumu 2013 (wet season)b [9]	n/a	Unclear	Yes	No	No	No	No	Yes	No	n/a	No
Okumu 2013 (wet season)c [9]	n/a	Unclear	Yes	No	No	No	No	Yes	No	n/a	No
Oxborough 2013 [49]	n/a	Unclear	Yes	No	No	No	No	Yes	Yes	Yes	No
Tungu 2010a [41]	n/a	Unclear	Yes	Yes	Yes	Yes	Yes	Yes	No	n/a	No
Tungu 2010b [41]	Unclear	Unclear	Yes	Yes	Yes	Yes	Yes	Yes	No	n/a	No
Tungu 2010c [41]	n/a	Unclear	Yes	Yes	Yes	Yes	Yes	Yes	No	n/a	No
Tungu 2010d [41]	Unclear	Unclear	Yes	Yes	Yes	Yes	Yes	Yes	No	n/a	No
Tungu 2010e [41]	Unclear	Unclear	Yes	Yes	Yes	Yes	Yes	Yes	No	n/a	No
Djenontin 2010 [34]	n/a	Unclear	Unclear	Yes	Yes	No	Yes	No	Yes	Yes	No

a Nets washed in accordance with WHO standardised protocol [24]. n/a indicates the net was unwashed.

b Huts cleaned and ventilated before the start of the study and after each rotation of net to prevent cross-contamination of insecticide.

c Bioassays using laboratory-reared mosquito populations conducted on ITNs before the study to ensure that impregnation of nets has been performed correctly.

d Bioassays using laboratory-reared mosquito populations conducted on ITNs at the end of the study to measure the residual activity.

e Chemical analysis of ITNs to ensure the correct dosage of insecticide is present.

f Resistance status of mosquito populations assessed using bioassay to measure the level of phenotypic resistance, *kdr* genotyping to measure the frequency of the L1014F or L1014S mutation, and metabolic resistance testing, which can be carried out using synergists, biochemical enzyme analysis, or gene expression profiling.

g n/a indicates *kdr* was not measured.

BFaso, Burkina Faso; M.ville, Malanville; n/a, not applicable; YFO, Yaokoffikro.

Characterisation of resistance was not consistent across studies. Seventeen comparisons measured phenotypic resistance using bioassays complemented with *kdr* genotyping in the mosquito populations under investigation. Bioassays on their own were used in 27 comparisons, whilst 11 comparisons were performed on mosquitoes for which only *kdr* genotyping was used. Characterisation of metabolic resistance was reported in just two studies, where the authors also measured phenotypic resistance and *kdr*. For those studies which screened for *kdr*, ten stated the number of mosquitoes that had been genotyped.

### Relationship between Resistance and Entomological Outcomes

#### Cone tests

Forty-seven cone test comparisons reported mosquito mortality (21 low, 20 moderate, and five high resistance and one unclear) (Figure S1). Mortality was very low in the untreated net group, and the risk of mosquito mortality is much higher using ITNs as compared with UTNs regardless of resistance. The study-specific RDs showed huge variability within all three categories of resistance. The meta-analytic results showed that the difference in mortality risk using ITNs as compared with UTNs decreased as resistance increased. Nevertheless, mortality risk was significantly higher for ITNs compared to UTNs regardless of resistance: with low resistance, the difference in risk of mortality is 0.86 (95% CI 0.72 to 1.01; 4,626 mosquitoes, 21 comparisons; *I* ^2^ = 100%, 95% CI 100% to 100%); in the case of moderate resistance the difference in risk is 0.71 (95% CI 0.53 to 0.88; 5,760 mosquitoes, 20 comparisons; *I* ^2^ = 100%, 95% CI 100% to 100%); with high resistance, the difference in risk is 0.56 (95% CI 0.17 to 0.95; 784 mosquitoes, five comparisons; *I* ^2^ = 99%, 95% CI 99% to 100%). The test for subgroup differences did not demonstrate a difference in the RD between high, medium, and low resistance subgroups (*p* = 0.12, *I* ^2^ = 49%, 95% CI 23% to 66%). A further 12 comparisons (seven low resistance, five high) presented data that could not be combined in meta-analysis (Table 11).

**Table 11:** Results from cone tests comparing LLIN or CTN versus UTN for mosquito mortality and knock-down at 60 min.

Study	Intervention (All versus UTN)	Net Washed	Mosquito Species (Strain)	Resistance Status	Mosquito Mortality			Knock-Down at 60 min		
					ITN (Percent)	UTN (Percent)	RD	ITN (Percent)	UTN (Percent)	RD
Koudou 2011 (Kisumu)a [42]	LLIN PermaNet 3.0	No	*An. gambiae* s.s. (Kisumu)	Low	99	0	0.99	98	0	0.98
Koudou 2011 (Kisumu)b [42]	LLIN PermaNet 3.0	Yes	*An. gambiae* s.s. (Kisumu)	Low	99	0	0.99	98	0	0.98
Koudou 2011 (Kisumu)c [42]	LLIN PermaNet 2.0	No	*An. gambiae* s.s. (Kisumu)	Low	100	0	1	99	0	0.99
Koudou 2011 (Kisumu)d [42]	LLIN PermaNet 2.0	Yes	*An. gambiae* s.s. (Kisumu)	Low	99	0	0.99	97	0	0.97
Koudou 2011 (Kisumu)e [42]	CTN deltamethrin 25 mg/m^2^	Yes	*An. gambiae* s.s. (Kisumu)	Low	95	0	0.95	95	0	0.95
Malima 2008 (cone)a [36]	LLIN Olyset	No	*An. gambiae* s.s. (Kisumu)	Low	99	0	0.99	75	0	0.75
Malima 2008 (cone)b [36]	CTN alpha-cypermethrin 20 mg/m^2^	No	*An. gambiae* s.s. (Kisumu)	Low	84	0	0.84	88	0	0.88
Koudou 2011 (YFO)a [42]	LLIN PermaNet 3.0	No	*An. gambiae* s.s. (Yaokoffikro wild population)	High	48	0	0.48	77	0	0.77
Koudou 2011 (YFO)b [42]	LLIN PermaNet 3.0	Yes	*An. gambiae* s.s. (Yaokoffikro wild population)	High	95	0	0.95	95	0	0.95
Koudou 2011 (YFO)c [42]	LLIN PermaNet 2.0	No	*An. gambiae* s.s. (Yaokoffikro wild population)	High	42	0	0.42	84	0	0.84
Koudou 2011 (YFO)d [42]	LLIN PermaNet 2.0	Yes	*An. gambiae* s.s. (Yaokoffikro wild population)	High	82	0	0.82	90	0	0.9
Koudou 2011 (YFO)e [42]	CTN deltamethrin 25 mg/m^2^	Yes	*An. gambiae* s.s. (Yaokoffikro wild population)	High	8	0	0.08	17	0	0.17

YFO, Yaokoffikro.

Nine comparisons reported percentage knock-down at 60 min (six low resistance, two high, one unclear; Figure S2). In mosquitoes with low resistance, the risk of being knocked down is significantly higher using ITNs as compared with UTNs, but with high resistance, there is no difference between ITNs and UTNs. A significant difference is detected between the meta-analytic results for mosquitoes with low, unclear, and high resistance (*p*<0.00001, *I* ^2^ = 98.8%, 95% CI 98.3% to 99.2%).

The majority of studies show that the risk of knock-down is higher using ITNs than using UTNs, regardless of resistance. In mosquitoes with low resistance, the difference in risk of knock-down is 0.87 (95% CI 0.69 to 1.05; 3,440 mosquitoes, 17 comparisons; *I* ^2^ = 100%, 95% CI 100% to 100%); with high resistance, the difference in risk is 0.09 (95% CI −0.03 to 0.21; 309 mosquitoes, two comparisons; *I* ^2^ = 87%, 95% CI 94% to 97%). There is high variability between the results from studies within the same resistance category, although all comparisons tend to favour ITNs. A further 12 comparisons (seven low resistance, five high) presented data that could not be combined in meta-analysis (Table 11).

Seven cone test comparisons reported time to 50% knock-down (four low resistance, three high), and two comparisons presented time to 95% knock-down (one low, one high). By visual inspection of Table 5, the knock-down times tend to be longer in studies of mosquitoes with high resistance than in studies of mosquitoes with low resistance. However, this comparison is made across trials and may be subject to confounding.

#### Tunnel tests

Fourteen tunnel test comparisons reported feeding (eight low resistance, two moderate, six high) (Figure S3). The higher the resistance, the lower the effectiveness of ITNs (as compared with UTNs). A significant difference is detected between the meta-analytic results for mosquitoes with low, moderate, and high resistance (*p* = 0.001, *I* ^2^ = 85.1%, 95% CI 68.7% to 92.9%). A lower risk of blood feeding is apparent when using ITNs as compared with UTNs, regardless of resistance. For mosquitoes with low resistance, the difference in the risk of blood feeding is −0.66 (95% CI −0.77 to −0.55; 2,177 mosquitoes, eight comparisons; *I* ^2^ = 92%, 95% CI 87% to 95%); for mosquitoes with moderate resistance, the difference in risk is −0.53 (95% CI −0.63 to −0.42; 300 mosquitoes, two comparisons; *I* ^2^ = 0% 95% CI not estimable); and for mosquitoes with high resistance, the difference in risk is −0.27 (95% CI −0.45 to −0.09; 2,472 mosquitoes, six comparisons; *I* ^2^ = 97%, 95% CI 94% to 98%). There is high variability among the results from studies of mosquitoes with low resistance and also among those from studies of mosquitoes with high resistance, although most comparisons significantly favour ITNs. Four additional comparisons (low resistance) presented data that could not be combined in meta-analysis (Table 6).

Sixteen tunnel test comparisons reported mosquito mortality (eight low resistance, two moderate, six high) (Figure S4). The risk of mortality is significantly higher using ITNs as compared with UTNs, regardless of resistance. The meta-analytic results showed that the difference in mortality risk using ITNs as compared with UTNs decreased as resistance increased. The test for subgroup differences showed significant variability between the meta-analytic results from low, moderate, and high resistance subgroups (*p* = 0.001, *I* ^2^ = 84.7%, 95% CI 67.9% to 92.7%). For mosquitoes with low resistance, the difference in risk is 0.74 (95% CI 0.61 to 0.87; 2,177 mosquitoes, eight comparisons; *I* ^2^ = 96%, 95% CI 94% to 97%); for mosquitoes with moderate resistance, the difference in risk is 0.50 (95% CI 0.40 to 0.60; 300 mosquitoes, two comparisons; *I* ^2^ = 11%, 95% CI not estimable); and for mosquitoes with high resistance, the difference in risk is 0.39 (95% CI 0.24 to 0.54; 2,472 mosquitoes, six comparisons; *I* ^2^ = 95%, 95% CI 94% to 98%). There is high variability among the results from studies of mosquitoes with low resistance and also among those from studies of mosquitoes with high resistance, yet almost all comparisons significantly favour ITNs. Table 6 shows the results of additional comparisons (low resistance) that could not be combined in meta-analysis.

Six tunnel test comparisons reported whether mosquitoes could not pass through the net (four low resistance, two high) (Figure S5). Results show that the higher the resistance, the lower the effectiveness of ITNs (as compared with UTNs). The observed trend could be caused by differences in characteristics (other than resistance) between the studies of low resistance mosquitoes and those of high resistance mosquitoes. A significant difference is detected between the meta-analytic results for low and high resistance mosquitoes (*p*<0.00001, *I* ^2^ = 98.4%, 95% CI 97.1% to 99.1%).

The risk of not passing though the net is significantly higher when using ITNs than when using UTNs, regardless of mosquito resistance. In mosquitoes with low resistance, the difference in risk is 0.68 (95% CI 0.62 to 0.75; 1,140 mosquitoes, four comparisons; *I* ^2^ = 61%, 95% CI 0% to 87%), and in mosquitoes with high resistance, the difference in risk is 0.36 (95% CI 0.31 to 0.41; 1,309 mosquitoes, two comparisons; *I* ^2^ = 0%, 95% CI not estimable). There is variability among the results from studies of mosquitoes with low resistance, yet all comparisons significantly favour ITNs. Four additional comparisons (low resistance) presented data that could not be combined in meta-analysis (Table 6).

#### Experimental hut trials

**Figure 2:**
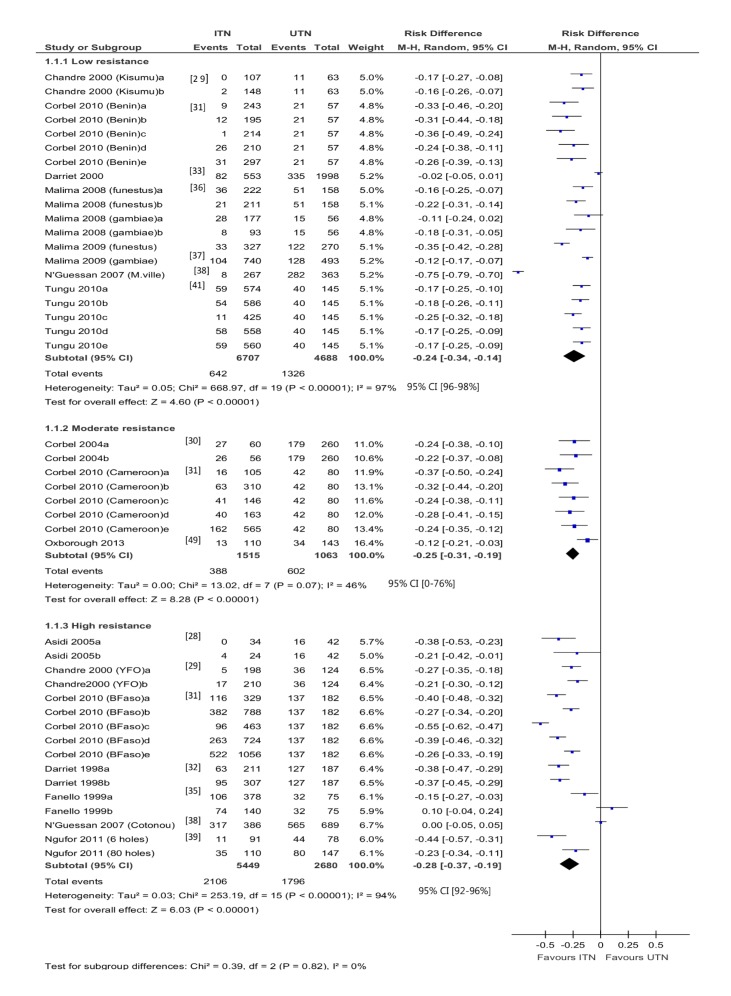
Forest plot for experimental hut trials comparing LLIN or CTN versus UTN for blood feeding. BFaso, Burkina Faso; M-H, Mantel-Haenszel; M.ville, Malanville (Benin); YFO, Yaokoffikro, (Côte d'Ivoire).

Overall, 44 hut trial comparisons reported blood feeding (20 low resistance, nine moderate, 15 high) (Figure 2). There is no clear relationship between resistance and the effectiveness of ITNs. A significant difference is not detected between the meta-analytic results for low, moderate, and high resistance groups (*p* = 0.84, *I* ^2^ = 0%, 95% CI 0% to 35%).

Blood feeding was reduced when using ITNs as compared with UTNs, regardless of resistance. In mosquitoes with low resistance, the difference in the risk of blood feeding is −0.24 (95% CI −0.34 to −0.14; 11,395 mosquitoes, 20 comparisons; *I* ^2^ = 97%, 95% CI 96% to 98%); in mosquitoes with moderate resistance, the difference in risk is −0.25 (95% CI −0.31 to −0.19; 2,578 mosquitoes, eight comparisons; *I* ^2^ = 46%, 95% CI 0% to 76%); and in mosquitoes with high resistance, the difference in risk is −0.28 (95% CI −0.37 to −0.19; 8,129 mosquitoes, 16 comparisons; *I* ^2^ = 94%, 95% CI 92% to 96%). There is particularly high variability among the results from studies of mosquitoes with low resistance and among those from studies of mosquitoes with high resistance, although most comparisons significantly favour ITNs. One comparison [22], with high resistance, reported 38% and 68% blood feeding (figures estimated from graph) in the ITN and UTN groups, respectively (RD = 0.3).

**Figure 3:**
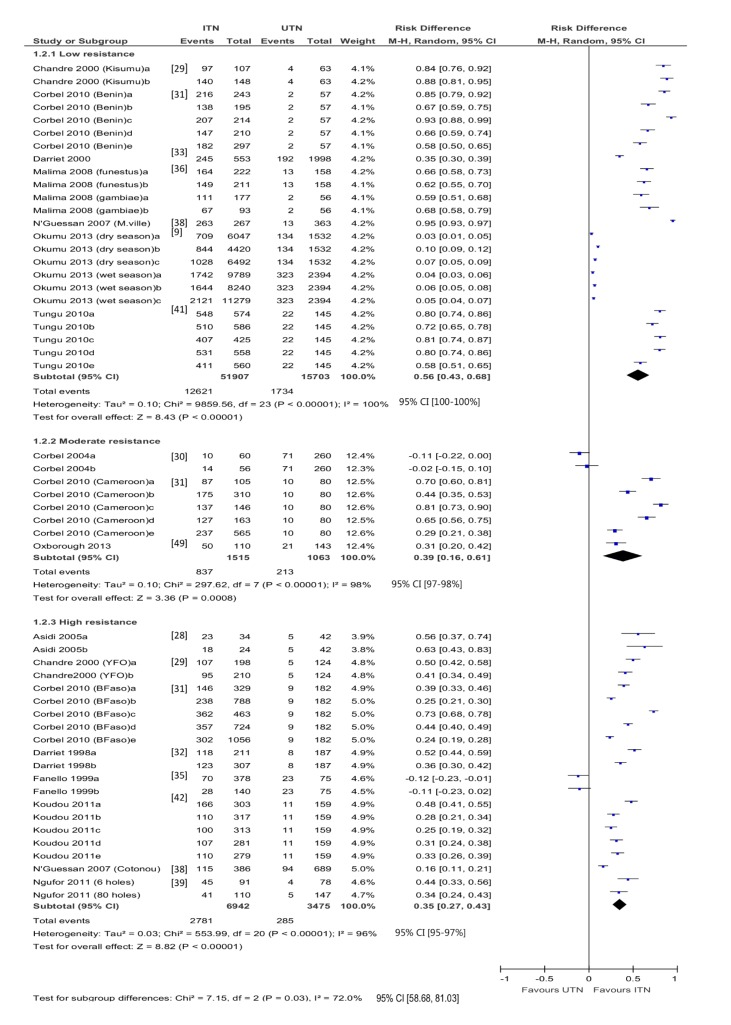
Forest plot for experimental hut trials comparing LLIN or CTN versus UTN for mosquito mortality. BFaso, Burkina Faso; M-H, Mantel-Haenszel; M.ville, Malanville (Benin); YFO, Yaokoffikro, (Côte d'Ivoire).

Fifty-three hut trial comparisons reported mosquito mortality (24 low resistance, eight moderate, 20 high) (Figure 3). There is high heterogeneity across study-specific results with each category of resistance. In addition, one study [9] appears to show no evidence of an effect of ITNs in low resistance mosquitoes. The authors also report on the bioassay, which shows 90%–100% susceptibility to insecticides. However, mortality risk was higher for ITNs compared to UTNs irrespective of the resistance category. In mosquitoes with low resistance, the difference in risk is 0.56 (95% CI 0.43 to 0.68; 67,610 mosquitoes, 24 comparisons; *I* ^2^ = 100%, 95% CI 100% to 100%); in mosquitoes with moderate resistance, the difference in risk is 0. 39 (95% CI 0.16 to 0.61; 2,578 mosquitoes, eight comparisons; *I* ^2^ = 98%, 95% CI 97% to 98%); and with high resistance, the difference in risk is 0.35 (95% CI 0.27 to 0.43; 10,417 mosquitoes, 21 comparisons; *I* ^2^ = 96%, 95% CI 95% to 97%). The meta-analytic results showed that the difference in mortality risk using ITNs as compared with UTNs modestly decreased as resistance increased, and the test for subgroup differences demonstrated a difference in the RD between high, medium, and low resistance subgroups (*p* = 0.03, *I* ^2^ = 72.0%, 95% CI 58.7% to 81.0%).

One comparison [22], with high resistance mosquitoes, reported 42% and 2% mortality (figures estimated from graph) in the ITN and UTN groups, respectively (RD = 0.4).

**Figure 4:**
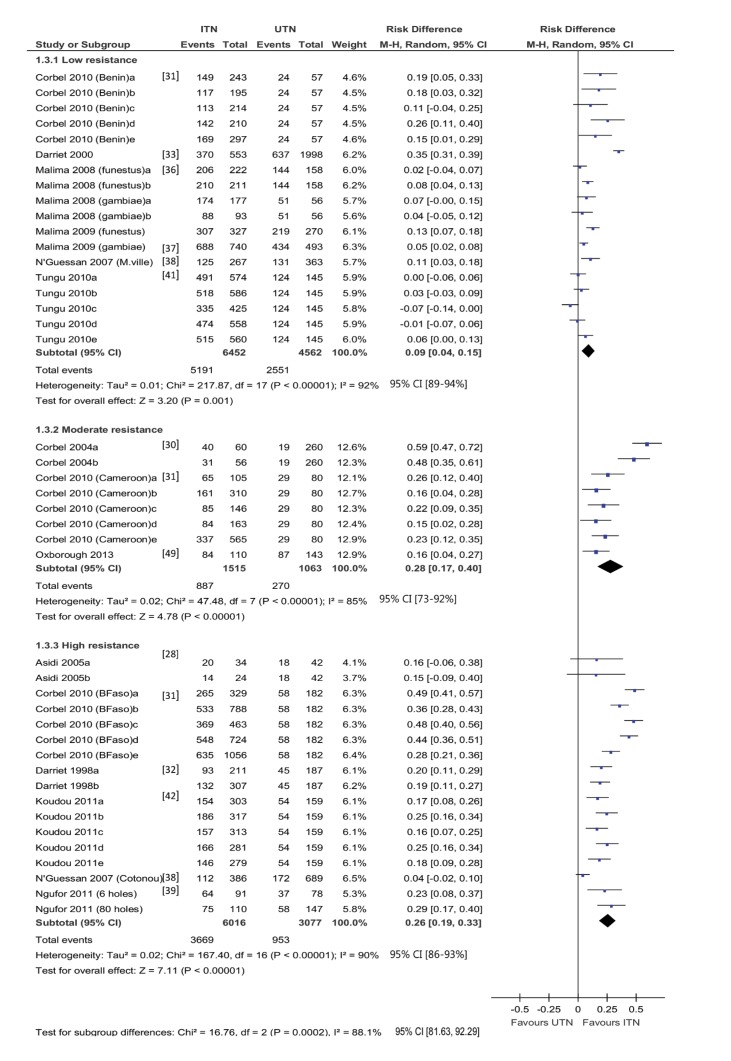
Forest plot for experimental hut trials comparing LLIN or CTN versus UTN for induced exophily. BFaso, Burkina Faso; M-H, Mantel-Haenszel; M.ville, Malanville (Benin); YFO, Yaokoffikro, (Côte d'Ivoire).

Forty-three trial hut comparisons reported results for induced exophily (18 low resistance, nine moderate, 16 high) (Figure 4). There is no clear relationship between resistance and the effectiveness of ITNs in relation to this outcome. A significant difference is detected between the meta-analytic results for low, moderate, and high resistance (*p* = 0.0002, *I* ^2^ = 88.2%, 95% CI 81.6% to 92.3%).

Generally, the risk of exiting the hut is higher using ITNs than using UTNs, regardless of resistance. For mosquitoes with low resistance, the difference in risk is 0.09 (95% CI 0.04 to 0.15; 11,014 mosquitoes, 18 comparisons; *I* ^2^ = 92%, 95% CI 89% to 94%); for mosquitoes with moderate resistance, the difference in risk is 0.28 (95% CI 0.17 to 0.40; 2,578 mosquitoes, eight comparisons; *I* ^2^ = 85%, 95% CI 73% to 92%); and for mosquitoes with high resistance, the difference in risk is 0.26 (95% CI 0.19 to 0.33; 8,695 mosquitoes, 16 comparisons; *I* ^2^ = 90%, 95% CI 86% to 93%). There is substantive heterogeneity within and across resistance groups, but most comparisons significantly favour ITNs. One comparison [22], with high resistance mosquitoes, reported 80% and 20% induced exophily (figures estimated from graph) in the ITN and UTN groups, respectively (RD = 0.6).

Fifty-five comparisons reported on deterrence (21 low resistance, 13 moderate, 21 high) (Table 12). There is no clear relationship between resistance status and deterrence based on a visual inspection of the results.

**Table 12:** Results from experimental hut trials comparing LLIN or CTN versus UTN for deterrence.

Study	Intervention (All versus UTN)	Net Washed	Mosquito Species	Total Number in ITN Huts	Resistance Status	Total Number in UTN Huts	Deterrence (Percent)
Chandre 2000 (Kisumu)a [29]	CTN deltamethrin 25 mg/m^2^	No	*An. gambiae* s.s.	107	Low	126	15
Chandre 2000 (Kisumu)b [29]	CTN permethrin 500 mg/m^2^	No	*An. gambiae* s.s.	148	Low	126	−17
Darriet 2000 [33]	CTN deltamethrin 25 mg/m^2^	No	*An. gambiae* s.s.	553	Low	1,998	72
Malima 2008 (funestus)a [36]	LLIN Olyset	No	*An. funestus*	222	Low	315	30
Malima 2008 (funestus)b [36]	CTN alpha-cypermethrin 20 mg/m^2^	No	*An. funestus*	211	Low	315	33
Malima 2008 (gambiae)a [36]	LLIN Olyset	No	*An. gambiae* s.s.	177	Low	112	−58
Malima 2008 (gambiae)b [36]	CTN permethrin 500 mg/m^2^	No	*An. gambiae* s.s.	93	Low	112	17
Malima 2009 (funestus) [37]	CTN deltamethrin 25 mg/m^2^	No	*An. funestus*	327	Low	270	−21
Malima 2009 (gambiae) [37]	CTN deltamethrin 25 mg/m^2^	No	*An. gambiae* s.s.	740	Low	493	−50
N'Guessan 2007 (M.ville) [38]	CTN lambda-cyalothrin 18 mg/m^2^	No	*An. gambiae* s.s.	267	Low	363	26
Tungu 2010a [41]	LLIN PermaNet 2.0	No	*An. gambiae* s.s.	574	Low	723	21
Tungu 2010b [41]	LLIN PermaNet 2.0	Yes	*An. gambiae* s.s.	586	Low	723	19
Tungu 2010c [41]	LLIN PermaNet 3.0	No	*An. gambiae* s.s.	425	Low	723	41
Tungu 2010d [41]	LLIN PermaNet 3.0	Yes	*An. gambiae* s.s.	558	Low	723	23
Tungu 2010e [41]	CTN deltamethrin 25 mg/m^2^	Yes	*An. gambiae* s.s.	560	Low	723	23
Okumu 2013 (dry season)a [9]	LLIN Olyset	No	*An. arabiensis*	6,047	Moderate	4,596	−32
Okumu 2013 (dry season)b [9]	LLIN PermaNet 2.0	No	*An. arabiensis*	4,420	Moderate	4,596	4
Okumu 2013 (dry season)c [9]	LLIN Icon Life (deltamethrin 65 mg/m^2^)	No	*An. arabiensis*	6,492	Moderate	4,596	−41
Okumu 2013 (wet season)a [9]	LLIN Olyset	No	*An. arabiensis*	9,789	Moderate	7,181	−36
Okumu 2013 (wet season)b [9]	LLIN PermaNet 2.0	No	*An. arabiensis*	8,240	Moderate	7,181	−15
Okumu 2013 (wet season)c [9]	LLIN Icon Life	No	*An. arabiensis*	11,279	Moderate	7,181	−57
Corbel 2004a [30]	CTN permethrin 500 mg/m^2^	No	*An. gambiae* s.s.	60	Moderate	520	88
Corbel 2004b [30]	CTN permethrin 250 mg/m^2^	No	*An. gambiae* s.s.	56	Moderate	520	89
Corbel 2010 (Benin)a [31]	LLIN PermaNet 2.0	No	*An. gambiae* s.s.	243	Moderate	285	15
Corbel 2010 (Benin)b [31]	LLIN PermaNet 2.0	Yes	*An. gambiae* s.s.	195	Moderate	285	32
Corbel 2010 (Benin)c [31]	LLIN PermaNet 3.0	No	*An. gambiae* s.s.	214	Moderate	285	25
Corbel 2010 (Benin)d [31]	LLIN PermaNet 3.0	Yes	*An. gambiae* s.s.	210	Moderate	285	26
Corbel 2010 (Benin)e [31]	CTN deltamethrin 25 mg/m^2^	Yes	*An. gambiae* s.s.	297	Moderate	285	−4
Corbel 2010 (Cameroon)a [31]	LLIN PermaNet 2.0	No	*An. gambiae* s.s.	105	Moderate	401	74
Corbel 2010 (Cameroon)b [31]	LLIN PermaNet 2.0	Yes	*An. gambiae* s.s.	310	Moderate	401	23
Corbel 2010 (Cameroon)c [31]	LLIN PermaNet 3.0	No	*An. gambiae* s.s.	146	Moderate	401	64
Corbel 2010 (Cameroon)d [31]	LLIN PermaNet 3.0	Yes	*An. gambiae* s.s.	163	Moderate	401	59
Corbel 2010 (Cameroon)e [31]	CTN deltamethrin 25 mg/m^2^	Yes	*An. gambiae* s.s.	565	Moderate	401	−41
Oxborough 2013 [49]	CTN alpha-cypermethrin 25 mg/m^2^	No	*An. arabiensis*	110	Moderate	143	23
Asidi 2005a [28]	CTN lambda-cyalothrin 18 mg/m^2^	No	*An. gambiae* s.s.	34	High	83	59
Asidi 2005b [28]	CTN lambda-cyalothrin 18 mg/m^2^	Yes	*An. gambiae* s.s.	24	High	83	71
Chandre 2000 (YFO)a [29]	CTN deltamethrin 25 mg/m^2^	No	*An. gambiae* s.s.	198	High	247	20
Chandre 2000 (YFO)b [29]	CTN permethrin 500 mg/m^2^	No	*An. gambiae* s.s.	210	High	247	15
Corbel 2010 (BFaso)a [31]	LLIN PermaNet 2.0	No	*An. gambiae* s.s.	329	High	908	64
Corbel 2010 (BFaso)b [31]	LLIN PermaNet 2.0	Yes	*An. gambiae* s.s.	788	High	908	13
Corbel 2010 (BFaso)c [31]	LLIN PermaNet 3.0	No	*An. gambiae* s.s.	463	High	908	49
Corbel 2010 (BFaso)d [31]	LLIN PermaNet 3.0	Yes	*An. gambiae* s.s.	724	High	908	20
Corbel 2010 (BFaso)e [31]	CTN deltamethrin 25 mg/m^2^	Yes	*An. gambiae* s.s.	1,056	High	908	−16
Darriet 1998a [32]	CTN deltamethrin 25 mg/m^2^	No	*An. gambiae* s.s.	211	High	373	43
Darriet 1998b [32]	CTN permethrin 500 mg/m^2^	No	*An. gambiae* s.s.	307	High	373	18
Fanello 1999a [35]	CTN alpha-cypermethrin 20 mg/m^2^	No	*An. gambiae* s.s.	378	High	149	−154
Fanello 1999b [35]	CTN etofenprox 200 mg/m^2^	No	*An. gambiae* s.s.	140	High	149	6
Koudou 2011b [42]	LLIN PermaNet 2.0	No	*An. gambiae* s.s.	317	High	796	60
Koudou 2011d [42]	LLIN PermaNet 2.0	Yes	*An. gambiae* s.s.	281	High	796	64
Koudou 2011a [42]	LLIN PermaNet 3.0	No	*An. gambiae* s.s.	303	High	796	62
Koudou 2011c [42]	LLIN PermaNet 3.0	Yes	*An. gambiae* s.s.	313	High	796	60
Koudou 2011e [42]	CTN deltamethrin 25 mg/m^2^	Yes	*An. gambiae* s.s.	279	High	796	64
N'Guessan 2007 (Cotonou) [38]	CTN lambda-cyalothrin 18 mg/m^2^	No	*An. gambiae* s.s.	386	High	689	44
Ngufor 2011 (6 holes) [39]	LLIN deltamethrin 55 mg/m^2^, 6 holes	No	*An. gambiae* s.s.	91	High	78	−17
Ngufor 2011 (80 holes) [39]	LLIN deltamethrin 55 mg/m^2^, 80 holes	No	*An. gambiae* s.s.	110	High	147	25

BFaso, Burkina Faso; M.ville, Malanville; YFO, Yaokoffikro.

### Results of Subgroup Analyses, Sensitivity Analyses, and Funnel Plots

Considerable heterogeneity was found across all studies, therefore sources of heterogeneity were explored using subgroup analyses. We carried out subgroup analyses by net type, insecticide used, the concentration of insecticide, and whether nets were washed or not. Because of the wide variation between studies in relation to these factors, these plots were numerous. We carried out analyses grouping in different ways, but these analyses did not provide any explanation of the heterogeneity between studies. The funnel plots do not resemble symmetric funnels; this may be because of the high level of variability between studies and the low quality of the evidence (see Figures S6–S13). For experimental hut trials, similar conclusions are drawn from the sensitivity analyses and primary analyses (Table S9; Figures S14–S20).

## Discussion

The study set out to determine whether mosquito resistance to insecticides is having an impact on entomological outcomes in ITNs compared to UTNs in three experimental settings: highly controlled cone studies, laboratory tunnel studies with animal bait, and field trials in huts with humans as the attractant. Cone tests for mosquito knock-down showed reduced levels of knock-down associated with higher levels of resistance. Laboratory tunnel test results demonstrated a reduced effect of ITNs in mosquitoes with higher levels of resistance in terms of blood feeding, mosquito mortality, and passage through the nets.

In experimental hut trials the RD for mortality for ITNs compared to UTNs showed that ITNs continued to have an effect in all categories of resistance. The meta-analytic results showed that the difference in mortality risk using ITNs as compared with UTNs modestly decreased as resistance increased, and the test for subgroup differences demonstrated a difference in the RD between high, medium, and low resistance subgroups. The substantive heterogeneity in the studies' results and design may mask the true relationship between resistance and the RD, and the results need to be interpreted with caution.

What is clear from the results is that ITNs continue to have a substantive effect compared to UTNs in many studies, and that despite best efforts, explaining the heterogeneity between studies has been problematic, with field studies showing quite varied results. Sometimes there are quite unexpected and inconsistent findings such as in the study by Okumu et al. [9], which shows no evidence of a benefit of insecticide despite bioassays indicating “sensitivity”. Studies overall are very poor in characterising the resistance pattern of the mosquitoes, and the classification systems are unclear and lack uniformity.

We observed a large amount of heterogeneity and bias across studies, which was particularly acute in the field studies. Variations in the wild mosquito populations—such as their resistance levels, age, blood feeding and mating status (factors that themselves could influence resistance levels and host-seeking behaviour)—and also the local environment cannot be controlled for across studies. In addition, the execution of the field trials was not uniform across the studies, e.g., washing of nets, rotation of nets/sleepers, season in which the trial took place, length of the trial, decontamination of huts, and exclusion of male mosquitoes from the analysis. Only one field trial conducted a direct comparison of susceptible versus resistant mosquitoes [29]. Deterrence could not be measured because the mosquitoes were directly placed inside the huts. For the remaining studies we conducted indirect comparisons between trials of nets in areas of high or moderate resistance and those in low resistance areas. Blinding of mosquito collectors, observers, and sleepers was not addressed in any of the studies.

One area of concern is that assessment of resistance of mosquito populations is not optimised across studies, and hence misclassification of resistance is likely to occur, adding to the high levels of heterogeneity. It is possible that target-site and metabolic resistance exert a differential impact on LLIN effectiveness, but most studies fail to accurately assess the presence of metabolic resistance. Insecticide resistance profiling of mosquito populations was varied across all studies, with just under half of the field studies measuring phenotypic resistance or *kdr* frequency, two out of the 14 studies measuring both, and only one measuring phenotypic resistance, *kdr*, and metabolic resistance [50]. Phenotypic resistance, as measured by bioassays, is regarded as the first step in identifying resistance [51]. It is prudent to always carry out bioassays to establish resistance levels before implementing mechanistic studies (e.g., genotyping for target-site and metabolic resistance and biochemical assays). It is unwise to assume that *kdr* alone is solely responsible for the resistant phenotype [52],[53]; mosquitoes could still harbour metabolic resistance, for example. Based on this, we were reluctant to label mosquito populations with no or low *kdr* frequency as “susceptible” (low resistance).

It is becoming increasingly clear that metabolic resistance often underpins pyrethroid resistance in mosquitoes, as demonstrated by both gene expression studies of resistant populations [11],[17],[18],[19],[20],[54] and enzyme characterisation studies [55],[56]. To date, resistance has been directly implicated in operational control failure of pyrethroids only in *An. funestus* in South Africa [57]. Metabolic resistance is the underlying mechanism [54],[58],[59], and therefore this mosquito species offers a unique opportunity to measure the impact of resistance on ITN efficacy. Unfortunately, none of the included studies have included the resistant form of this species.

A large number of studies were excluded because the insecticide resistance status of the wild mosquito populations was not characterised at the time of the study, but rather relied upon retrospective data. Mosquito populations are dynamic, and although a *kdr* frequency of >0.90, which is close to fixation, is unlikely to revert rapidly, we cannot rule out the migration of mosquito populations or other confounding factors that could dramatically influence mosquito populations and/or resistance profiles over time.

In terms of interpreting the patterns, this has to be done with care, given the variability of the results. Reduced killing of mosquitoes with increasing resistance in tunnel and hut studies raises concerns. Feeding preferences of mosquitoes can be plastic [60], and there is evidence that anthropogenic species such as *An. gambiae* and *An. funestus* can switch to feeding on cattle to obtain a blood meal in the presence of pyrethroid-treated materials [61],[62]. So, although the personal protection properties of ITNs (i.e., prevention of blood feeding and induced exophily) are still maintained, there is still the risk that if different hosts are available, mosquitoes could adapt their feeding preferences and thereby maintain large population sizes. If LLIN coverage is lowered, nets become badly damaged, are inappropriately used, are sold on, or are used less over time (all of which are realistic scenarios) [63], the reduced killing of resistant mosquitoes, which may have obtained a blood meal elsewhere, could be a cause for concern.

Inconsistency between studies in relation to study design, execution, and reporting format across all experimental hut trials is an obstacle in addressing the relationship between resistance and ITN efficacy confidently. There are no clear guidelines for measuring ITN efficacy against resistant mosquitoes. As a consequence, the studies do not easily lend themselves to meta-analysis, and so it is difficult to generate a consensus. It is likely that the effects of resistance on some outcomes may be moderate or small, but the lack of standardisation means the methodological differences between studies obscure any detection or coherent synthesis between studies. So, if this field of research aims to identify generalisable findings, then researchers need to consider how best to measure the dependent and independent variables so that the results are more comparable. Our concern with this lack of transparency and standardisation, and the need for improved reporting, echoes recent calls [64] for research to be better planned, co-ordinated, and of higher quality. With such gaps and lack of standardisation in the primary studies, it could be argued that current research represents inefficient use of scarce resources of the scientific community as a whole.

Based on the studies included in this meta-analysis, ITNs remain at least somewhat effective against African anopheline mosquitoes even when resistance has developed. However, whether ITNs remain effective against resistant mosquitoes cannot be definitively addressed whilst the execution and reporting of field studies and the profiling of resistance in mosquito populations is inadequate and inconsistent. Ideally, phenotypic resistance, target-site resistance, and metabolic resistance testing should all be applied to mosquito populations in the vicinity of the hut trial. If this is not feasible, then a combination of either phenotypic and target-site resistance testing or target-site and metabolic resistance testing should be performed. Authors should make it clear in their reporting if they have omitted to test for any of the three categories of resistance highlighted above. It is also imperative that resistance is measured at the time of the study rather than relying on retrospective data. International agreement is needed for standardised methods for measuring the impact of resistance on ITNs before conclusive statements about the effect of resistance can be made. In order to initiate dialogue about the standardisation of methods and reporting we have generated a list of criteria that need to be addressed based on the experience of this review (Box 2). It is important that policy makers and non-governmental organizations plan vector control strategies and purchase ITNs based on the best available data.

**Box 2. Considerations for Experimental Hut Study Design and Reporting**

**Resistance Testing of Mosquito Populations: Reporting Information Required**

- Phenotypic resistance: doses of insecticide tested, exposure times to insecticide, total number of mosquitoes tested, total number of mosquitoes killed
- Target-site resistance: type of mutation screened for (i.e., L1014F or L104S), associated *kdr* allele frequencies
- Metabolic resistance: identification of genes or enzyme class implicated in conferring resistance

**Study Design Reporting Criteria: Reporting Requirement**

- Study start date: date
- Study duration: number of nights
- Mosquito species present at location: species name and molecular form
- Nets randomly allocated to huts at start of trial: yes or no
- Nets rotated between huts during trial: yes or no
- Sleepers rotated between huts during trial: yes or no
- Washing of nets: wash procedure provided
- Huts cleaned between rotations: yes or no
- Observers collecting mosquitoes blinded to intervention: yes or no
- Sleepers blinded to intervention: yes or no
- Male mosquitoes used in the analysis: excluded or included
- Raw data for measured outcomes: provided
- Raw data for UTNs: provided

## Supporting Information

Figure S1 **Forest plot for cone tests comparing LLIN or CTN versus UTN for mosquito mortality.**
(EPS)

Figure S2 **Forest plot for cone tests comparing LLIN or CTN versus UTN for knock-down at 60 min.**
(EPS)

Figure S3 **Forest plot for tunnel tests comparing LLIN or CTN versus UTN for mosquito mortality.**
(EPS)

Figure S4 **Forest plot for tunnel tests comparing LLIN or CTN versus UTN for blood feeding.**
(EPS)

Figure S5 **Forest plot for tunnel tests comparing LLIN or CTN versus UTN for not passed though net.**
(EPS)

Figure S6 **Funnel plot for mosquito mortality for cone tests.**
(EPS)

Figure S7 **Funnel plot for percentage knock-down at 60 min for cone tests.**
(EPS)

Figure S8 **Funnel plot for blood feeding for tunnel tests.**
(EPS)

Figure S9 **Funnel plot for mosquito mortality for tunnel tests.**
(EPS)

Figure S10 **Funnel plot for deterrence for tunnel tests.**
(EPS)

Figure S11 **Funnel plot for blood feeding for experimental hut trials.**
(EPS)

Figure S12 **Funnel plot for mosquito mortality for experimental hut trials.**
(EPS)

Figure S13 **Funnel plot for induced exophily for experimental hut trials.**
(EPS)

Figure S14 **Forest plot for sensitivity analysis for blood feeding in hut studies where ITNs were randomly allocated to huts.**
(PDF)

Figure S15 **Forest plot for sensitivity analysis for mosquito mortality in hut studies where ITNs were randomly allocated to huts.**
(PDF)

Figure S16 **Forest plot for sensitivity analysis for induced exophily in hut studies where ITNs were randomly allocated to huts.**
(PDF)

Figure S17 **Forest plot for sensitivity analysis for blood feeding in hut studies where ITNs were rotated between huts.**
(PDF)

Figure S18 **Forest plot for sensitivity analysis for mosquito mortality in hut studies where ITNs were rotated between huts.**
(PDF)

Figure S19 **Forest plot for sensitivity analysis for induced exophily in hut studies where ITNs were rotated between huts.**
(PDF)

Figure S20 **Forest plot for sensitivity analysis for blood feeding in hut studies where sleepers were rotated between huts.**
(PDF)

Figure S21 **Forest plot for sensitivity analysis for mosquito mortality in hut studies where sleepers were rotated between huts.**
(PDF)

Figure S22 **Forest plot for sensitivity analysis for induced exophily in hut studies where sleepers were rotated between huts.**
(PDF)

Protocol S1 **Protocol for the impact of pyrethroid resistance on the efficacy of insecticide treated bed nets against anopheline mosquitoes: systematic review.**
(DOCX)

Table S1 **Search terms for electronic databases.**
(XLSX)

Table S2 **Example of the form used to assess the eligibility of each study based on the inclusion criteria.**
(XLSX)

Table S3 **Example of the form used for data extraction for cone tests.**
(XLSX)

Table S4 **Example of the form used for data extraction for tunnel tests.**
(XLSX)

Table S5 **Example of the form used for data extraction for experimental hut trials.**
(XLSX)

Table S6 **Risk of bias assessment for the included cone tests.**
(XLSX)

Table S7 **Risk of bias assessment for the included tunnel tests.**
(XLSX)

Table S8 **Risk of bias assessment for the included experimental hut trials.**
(XLSX)

Table S9 **Summary of sensitivity analysis for hut studies with low risk of bias.**
(XLSX)

## Abbreviations

CTN: conventionally treated bed net
ITN: insecticide-treated bed net
*kdr*: knock-down resistance
LLIN: long-lasting insecticide-treated bed net
RD: risk difference
UTN: untreated bed net
WHO: World Health Organization
