# Supplementary figures and images for: The Impact of Pyrethroid Resistance on the Efficacy of Insecticide-Treated Bed Nets against African Anopheline Mosquitoes: Systematic Review and Meta-Analysis
Source: PLoS Med. 2014 Mar 18;11(3):e1001619. doi: 10.1371/journal.pmed.1001619 (PMC3958359; doi:10.1371/journal.pmed.1001619)

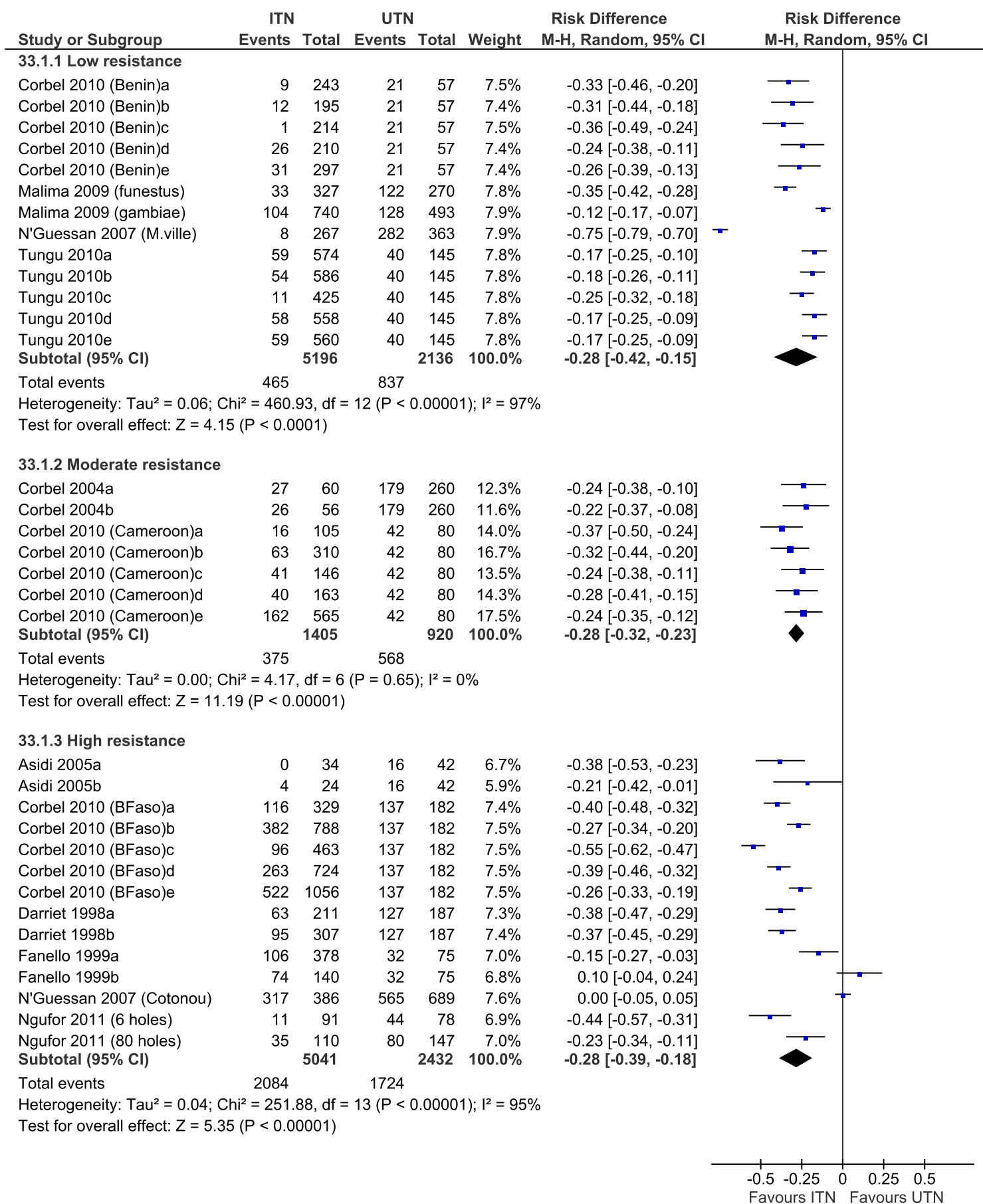

Test for subgroup differences:  $\chi^2 = 0.02$ ,  $df = 2$  ( $P = 0.99$ ),  $I^2 = 0\%$

Supplement: Figure S14 — Forest plot for sensitivity analysis for blood feeding in hut studies where ITNs were randomly allocated to huts. (PDF) [file pmed.1001619.s014.pdf]

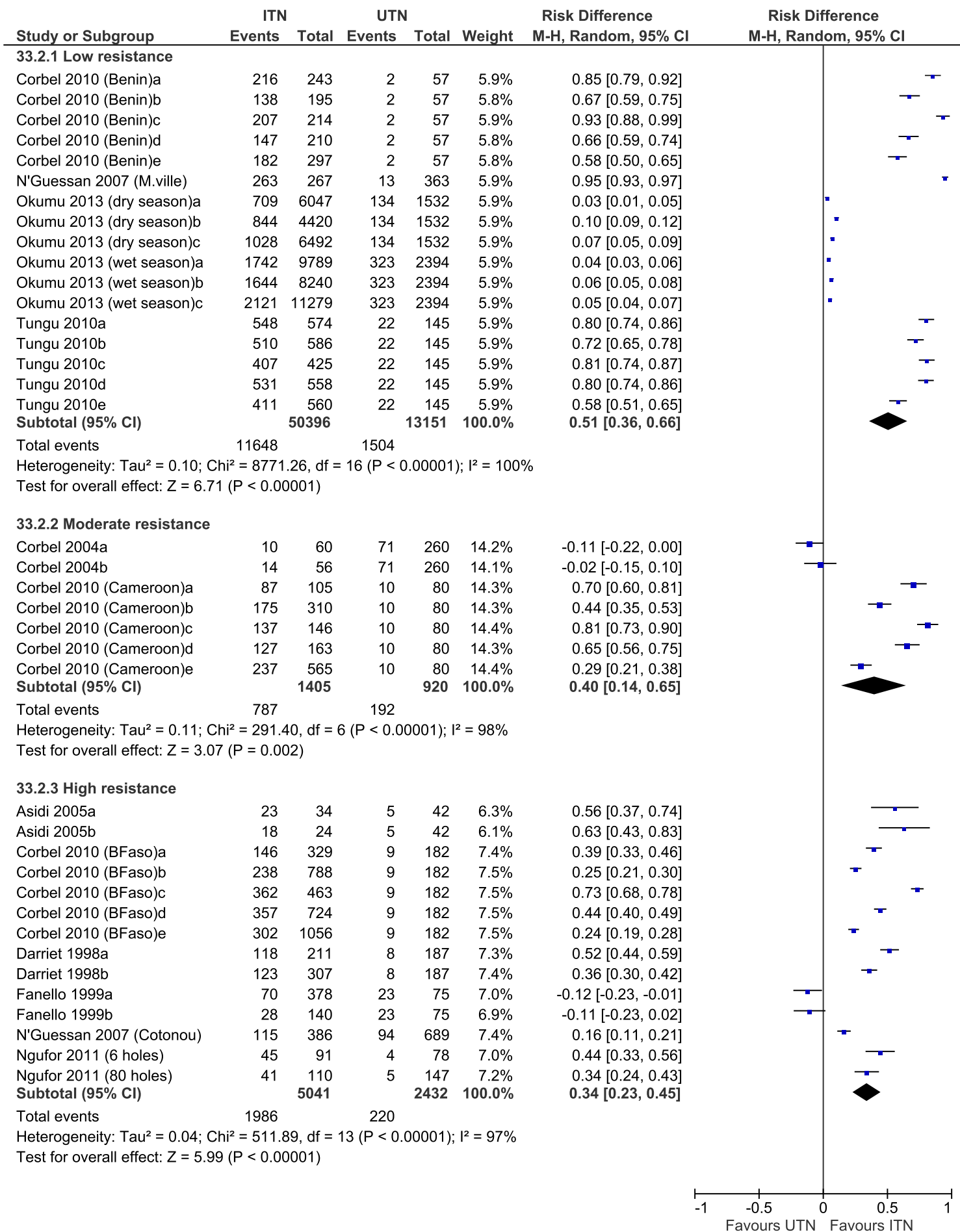

Test for subgroup differences:  $\chi^2 = 3.14$ ,  $df = 2$  ( $P = 0.21$ ),  $I^2 = 36.4\%$

Supplement: Figure S15 — Forest plot for sensitivity analysis for mosquito mortality in hut studies where ITNs were randomly allocated to huts. (PDF) [file pmed.1001619.s015.pdf]

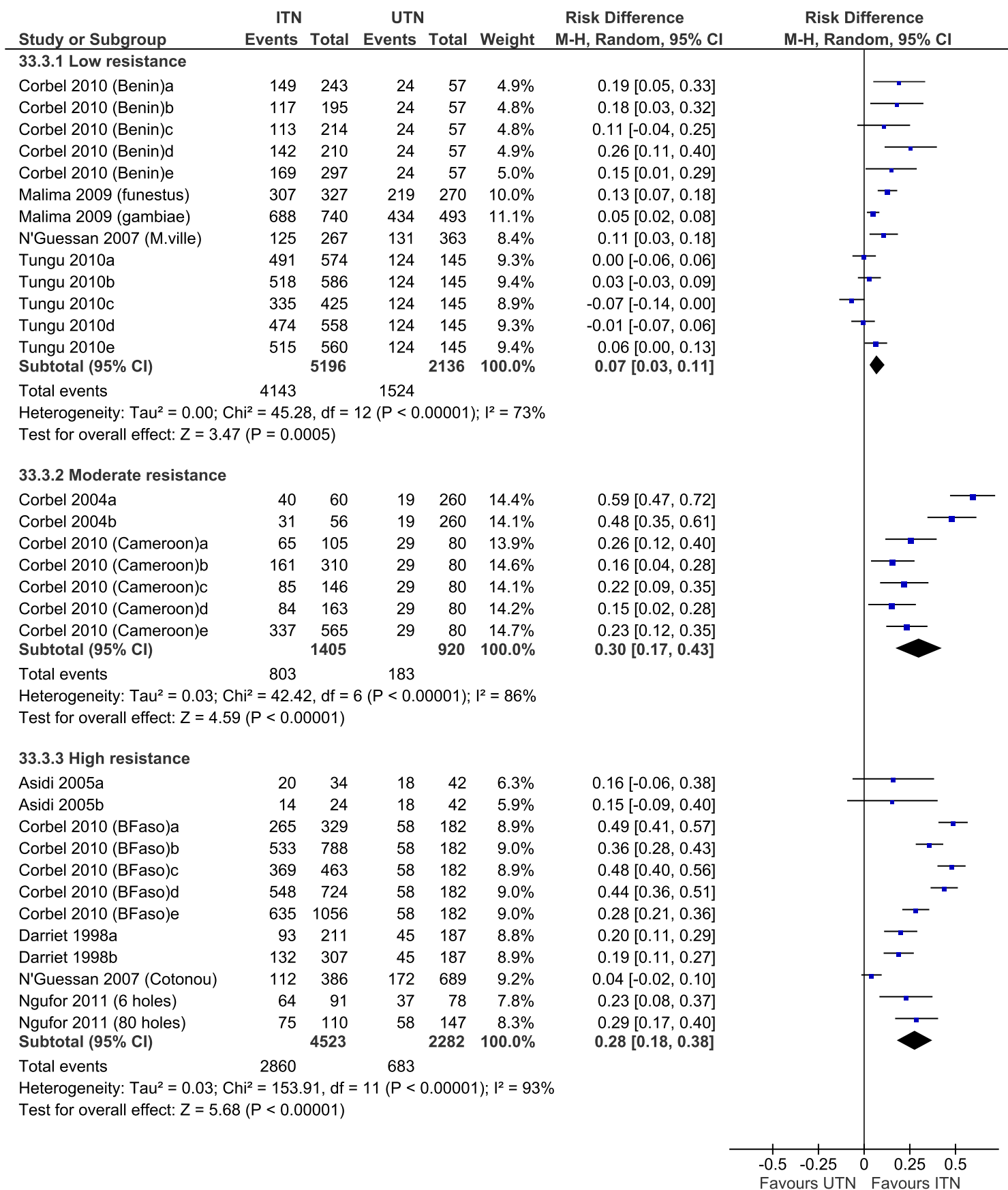

Test for subgroup differences:  $\chi^2 = 23.45$ ,  $df = 2$  ( $P < 0.00001$ ),  $I^2 = 91.5\%$

Supplement: Figure S16 — Forest plot for sensitivity analysis for induced exophily in hut studies where ITNs were randomly allocated to huts. (PDF) [file pmed.1001619.s016.pdf]

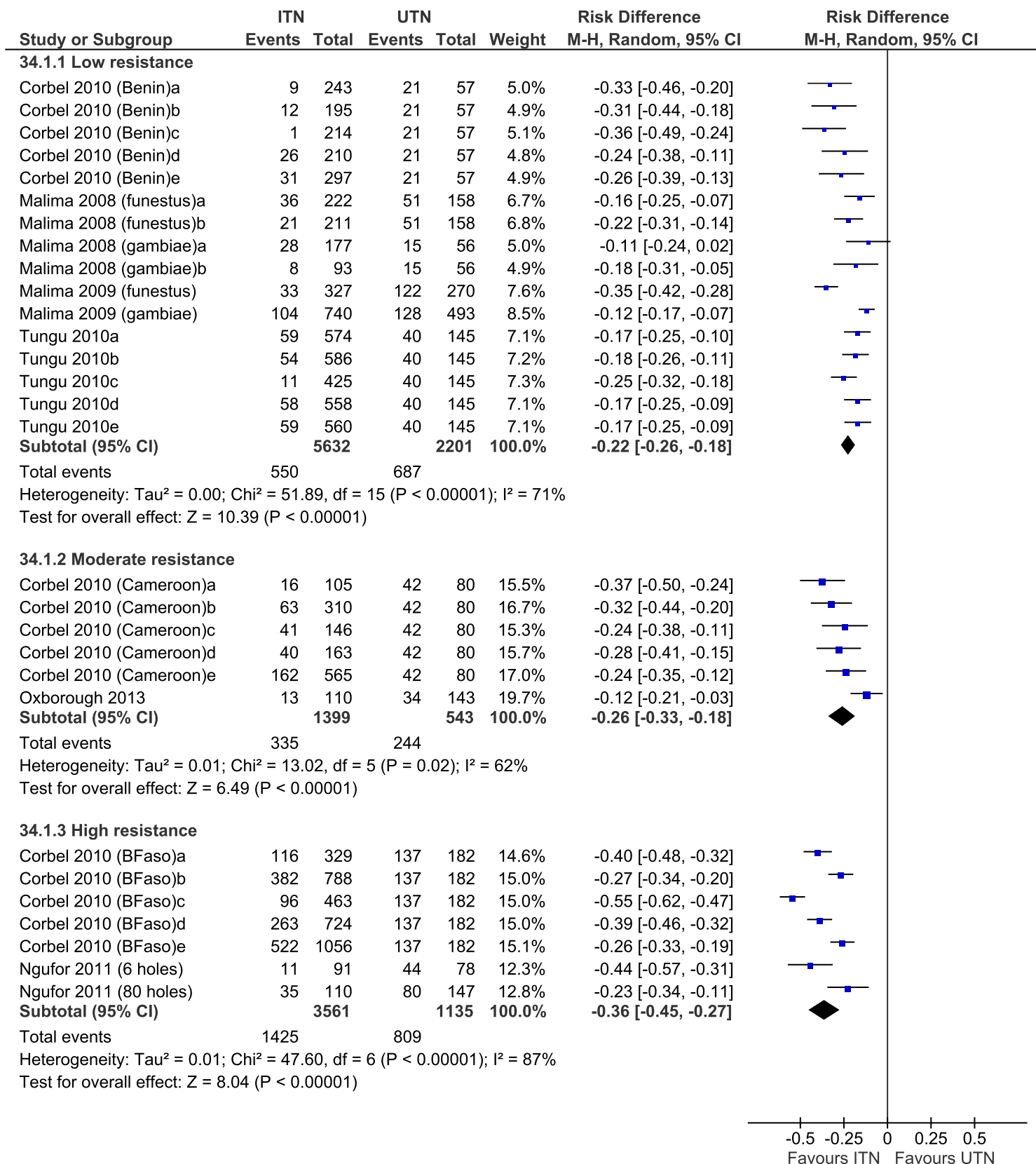

Test for subgroup differences:  $\chi^2 = 8.22$ ,  $df = 2$  ( $P = 0.02$ ),  $I^2 = 75.7\%$

Supplement: Figure S17 — Forest plot for sensitivity analysis for blood feeding in hut studies where ITNs were rotated between huts. (PDF) [file pmed.1001619.s017.pdf]

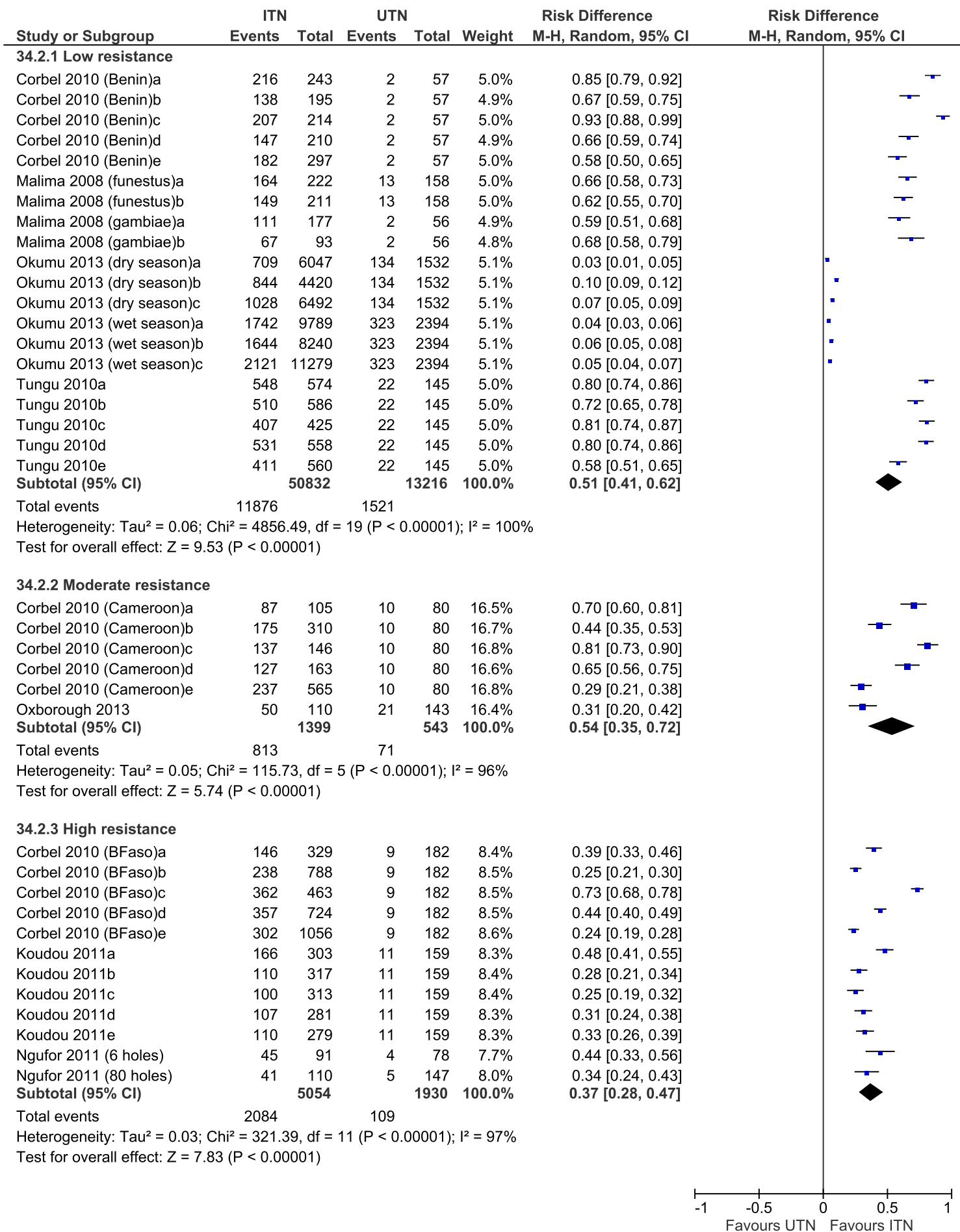

Test for subgroup differences:  $\chi^2 = 4.81$ ,  $df = 2$  ( $P = 0.09$ ),  $I^2 = 58.5\%$

Supplement: Figure S18 — Forest plot for sensitivity analysis for mosquito mortality in hut studies where ITNs were rotated between huts. (PDF) [file pmed.1001619.s018.pdf]

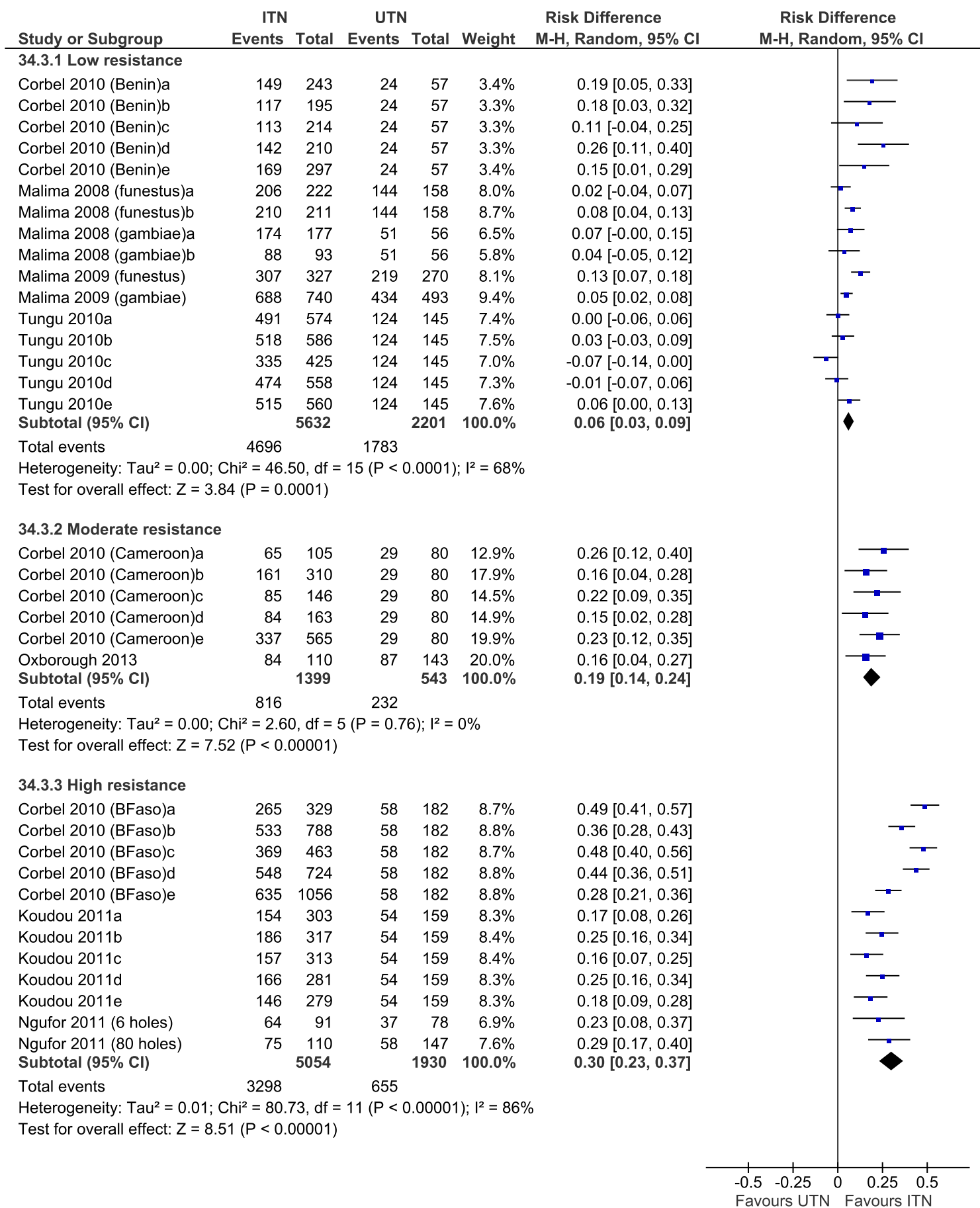

Test for subgroup differences:  $\chi^2 = 47.27$ ,  $df = 2$  ( $P < 0.00001$ ),  $I^2 = 95.8\%$

Supplement: Figure S19 — Forest plot for sensitivity analysis for induced exophily in hut studies where ITNs were rotated between huts. (PDF) [file pmed.1001619.s019.pdf]

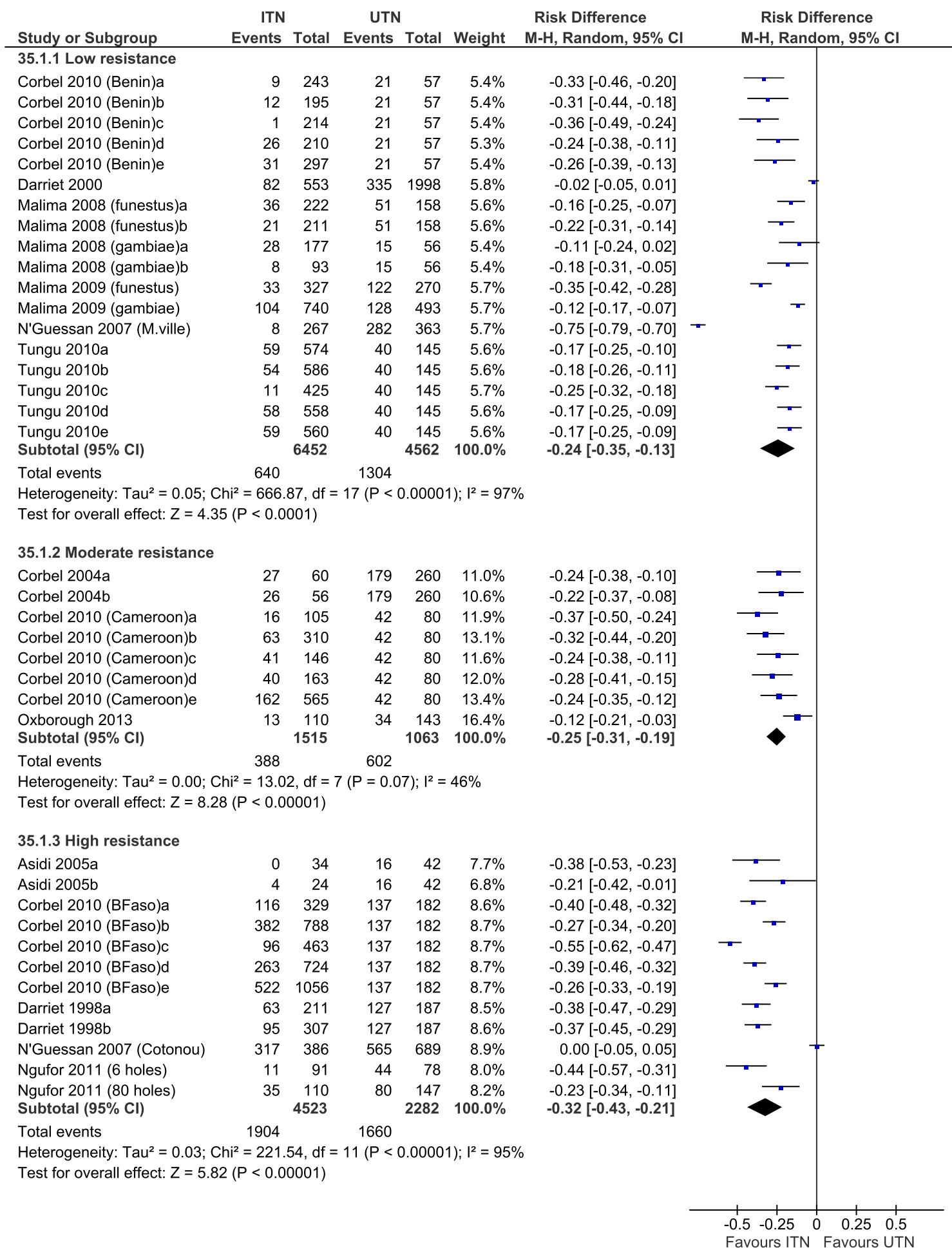

Test for subgroup differences: Chi<sup>2</sup> = 1.50, df = 2 (P = 0.47), I<sup>2</sup> = 0%

Supplement: Figure S20 — Forest plot for sensitivity analysis for blood feeding in hut studies where sleepers were rotated between huts. (PDF) [file pmed.1001619.s020.pdf]

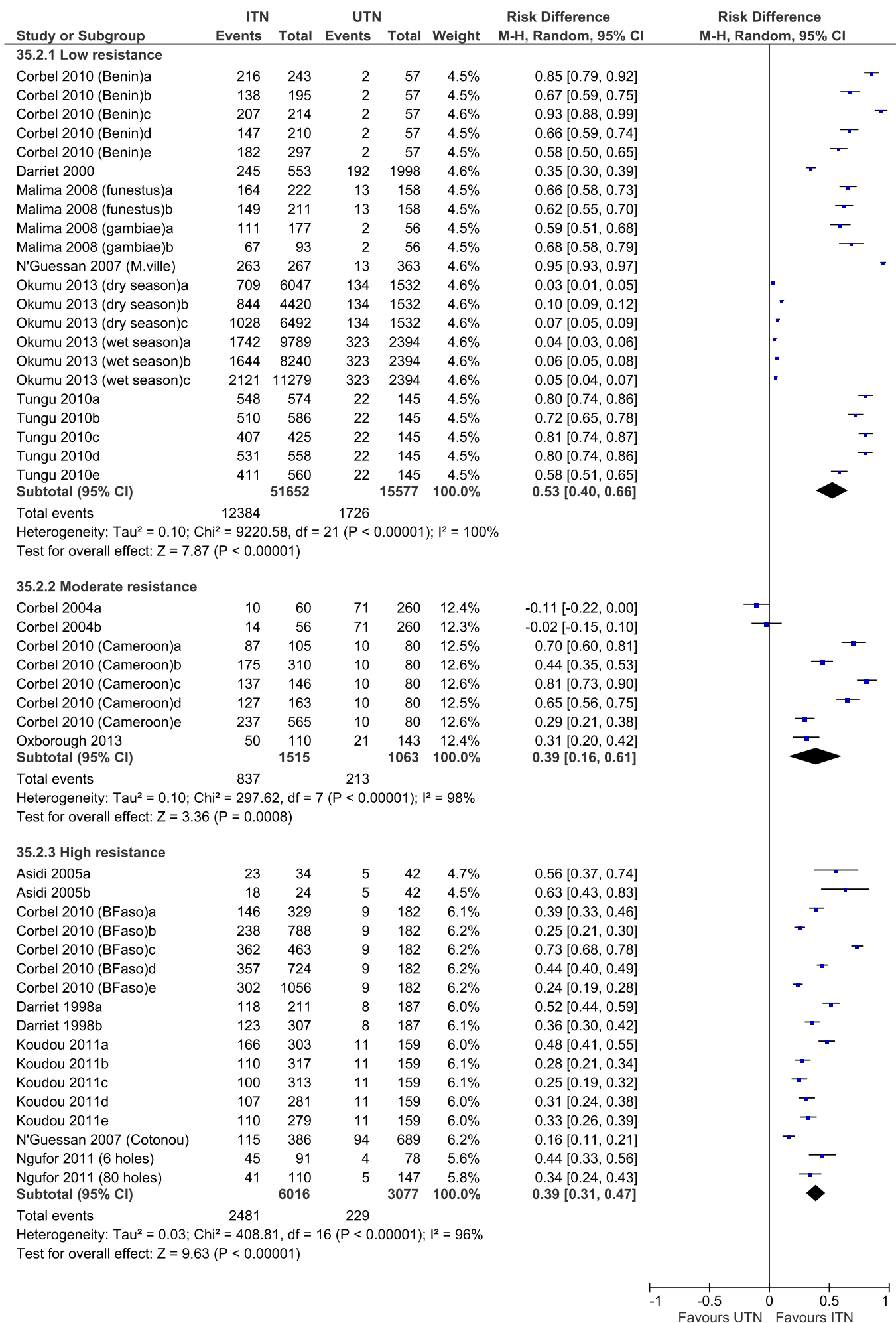

Supplement: Figure S21 — Forest plot for sensitivity analysis for mosquito mortality in hut studies where sleepers were rotated between huts. (PDF) [file pmed.1001619.s021.pdf]

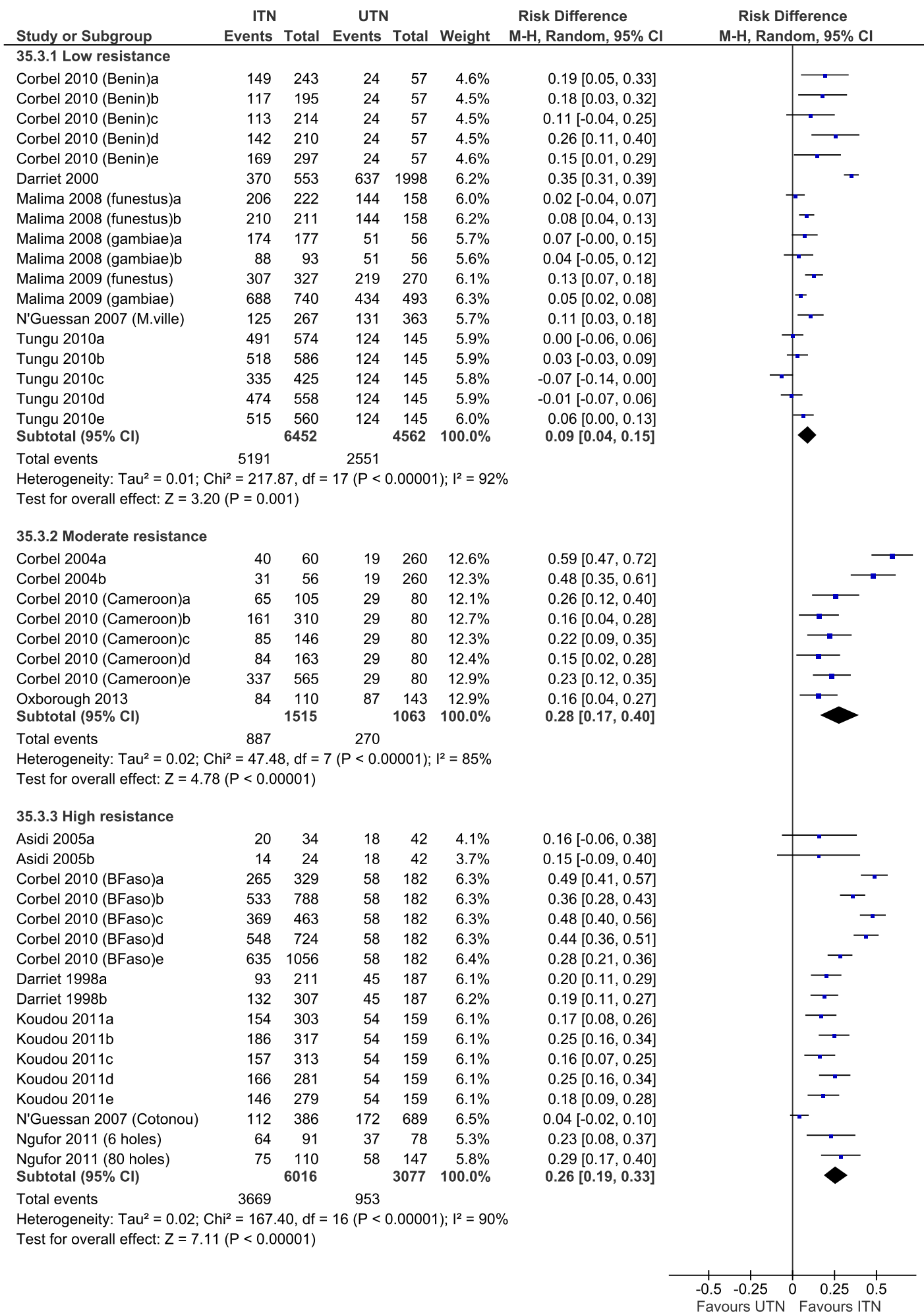

Supplement: Figure S22 — Forest plot for sensitivity analysis for induced exophily in hut studies where sleepers were rotated between huts. (PDF) [file pmed.1001619.s022.pdf]
