# Supplementary material for: The Impact of Pyrethroid Resistance on the Efficacy of Insecticide-Treated Bed Nets against African Anopheline Mosquitoes: Systematic Review and Meta-Analysis
Source: PLoS Med. 2014 Mar 18;11(3):e1001619. doi: 10.1371/journal.pmed.1001619 (PMC3958359; doi:10.1371/journal.pmed.1001619)
Supplement: Protocol S1 — Protocol for the impact of pyrethroid resistance on the efficacy of insecticide treated bed nets against anopheline mosquitoes: systematic review. (DOCX) [file pmed.1001619.s023.docx]

**Protocol S1:**

**Protocol for the impact of pyrethroid resistance on the efficacy of insecticide treated bed nets against Anopheline mosquitoes: systematic review**

Clare Strode, Sarah Donegan, Paul Garner, Ahmada Enyati and Janet Hemingway.

**Introduction**

The WHO estimate of the annual global death toll to malaria for 2010 was 655,000 with 86% of those deaths occurring in children under five years [[1](#_ENREF_1)]. An estimated 3.3 billion people were at risk from malaria in 2010. Whilst the annual risk of malaria is higher than that of previous years, the annual malaria death toll has declined by about 25% over the last ten years. This decline in mortality, particularly in Africa, can be attributed to the massive scaling up of control measures which have been driven by international donors. Along with indoor residual house spraying (IRS) and Artemisinin-based Combination Therapies (ACTs), insecticide treated nets (ITNs) form a major component of the WHO-led Roll Back Malaria campaign. ITNs use can significantly reduce malaria induced mortality and morbidity [[2](#_ENREF_2)]. In addition to offering personal protection to the user at scale they provide community wide protection by reducing the number of infective mosquitoes in the vicinity where ITNs are used. Between 2008 and 2010, 254 million ITNs were supplied to countries in Sub-Saharan Africa and the proportion of African households in possession of a net rose from 3% in 2000 to 50% by 2010 [[3](#_ENREF_3)].

ITNs exploit the anthropophagic and endophilic behaviour of the African vectors *Anopheles gambiae s.s.* and *An. funestus*. Nets, when in good condition and used correctly, are effective, simple to use, easy to deliver (eg. to rural communities) and compared with IRS less expensive and longer lasting. On account of their low mammalian toxicity, speed of action and high insecticidal activity pyrethroids are the only insecticide class recommended by WHO for use in ITNs. Two types of ITNs are currently available; conventionally treated nets (CTNs) (i.e. ones which require dipping into insecticide and which also require retreatment at least once a year) and the new generation long lasting insecticide nets (LLINs) which are factory-treated nets where the insecticide is incorporated within or bound around the net fibres. LLINs are required to retain their effective biological activity without re-treatment for at least 20 WHO standard washes under laboratory conditions and three years of recommended use under field conditions. The WHO Global Malaria Programme has instructed national malaria control programmes and their partners involved in ITN interventions to only purchase long-lasting insecticidal nets (LLINs) rather than CTNs.

Unfortunately the success and sustainability of ITNs and IRS are threatened by the emergence and spread of insecticide resistance to all four classes of insecticides available for public health (pyrethroids, organochlorides, organophosphates and carbamates). Currently 27 countries in Sub-Saharan Africa have reported pyrethroid resistance in Anopheles vectors [[4](#_ENREF_4)]. This figure could very well be higher but a lack of in-country resistance monitoring prevents accurate assessment. Because of their pyrethroid dependency, ITNs are especially vulnerable to insecticide resistance, as unlike IRS there are no readily available alternative insecticides. This has led WHO to suggest that pyrethroids should not be used for IRS in areas with high LLIN coverage [[1](#_ENREF_1)] to prevent amplifying pyrethroid resistance.

Insecticide resistance is multifaceted incorporating target-site, metabolic, cuticular resistance and behavioural avoidance. Target site resistance to pyrethroids in *An. gambiae* and An. arabiensis is underpinned by non-silent point mutations in the sodium channel gene (either L1014F or L1014S), which is referred to as knock-down resistance (*kdr*) [[5](#_ENREF_6),[6](#_ENREF_7)]. Target-site resistance prevents the successful binding of the insecticide molecule to the nerve membrane. Metabolic resistance is caused by the activity of three large multi gene families (cytochrome P450s, glutathione transferases and carboxylesterases) which are able to metabolise or sequester the insecticide thereby preventing it from reaching its target [[7](#_ENREF_8)]. It is becoming clear that the cytochrome P450s are responsible for the majority of cases of metabolic resistance with a secondary role for the glutathione transferases [[8](#_ENREF_9),9,[10](#_ENREF_11)]. There is also tantalising evidence that cuticular resistance may be a contributing factor, but this aspect requires further analysis [[8](#_ENREF_9),9 ,[11](#_ENREF_13)]. As pyrethroids and the organochlorine insecticide DDT target the sodium channel gene, cross resistance to both insecticides is common.

Understandably there is growing concern that increasing pyrethroid resistance could nullify the effectiveness of ITNs, resulting in malaria control failure on an operational scale [[12](#_ENREF_14)]. This worry is compounded by the fact that there are no readily available replacement insecticides for pyrethroids on LLINs. In a recent study the extensive deployment and use of 6 million LLINs has been blamed for selecting resistance in Anopheles vectors in Senegal where malaria morbidity also increased [[13](#_ENREF_15)].

Researchers have investigated whether the presence of resistance in mosquitoes attenuates the effects of pyrethroids on entomological outcomes. The literature is diverse, includes observational field studies, and laboratory experiments. However, there remains debate around the size of the effect, and whether ITNs remain effective. In order to inform this important policy question, we will carry out a systematic review of all relevant studies. Furthermore we intend to describe the range of methods and outcomes used across different studies.

**Objective**

To determine whether insecticide resistance in African Anopheline mosquitoes attenuates the effects of ITNs in terms of entomological outcomes and if so determine the size of the effects.

**Methods**

**Inclusion criteria –** studies which meet the following criteria will be considered for inclusion in the review;

1. **Study design:**
   1. Field trials using experimental huts
   2. Laboratory studies (cone tests and tunnel tests)
2. **Mosquito population:** African malaria vectors (*i.e.* *An. gambiae, An. arabiensis* or *An. funestus*). It is important that in the case of experimental hut studies using wild mosquitoes the investigators should have measured the resistance status of the mosquitoes at the time of the study. This can be done by bioassays and/or genotyping. In the case of lab studies, established strains of known genotype and/or phenotype will be considered for inclusion.
3. **Types of Intervention:** An ITN (CTN or a LLIN) that has been compared against an untreated bed net (UTN) which serves as a control. The ITNs must have been impregnated with a WHO recommended pyrethroid with the recommended formulation and dose (Table S1 for CTNs and Table S2 for LLINs).
4. **Outcomes:** The types of outcomes included will be based on the type of study.

- Experimental huts studies;
  - blood feeding
  - mosquito mortality
  - deterrence
  - induced exophily (number found in exit trap),
- Lab studies (tunnel tests)
  - not passed though net
  - blood feeding
  - mosquito mortality
- Lab studies (cone tests)
  - mosquito mortality
  - knock down at 60 minutes
  - time to 50% knock down
  - Time to 95% knock down

**Search strategy**

The following databases will be accessed to identify relevant studies;

1. MEDLINE
2. Cochrane Central register of Controlled Trials (CENTRAL)
3. Science Citation Index Expanded (SCI-EXPANDED)
4. Social Sciences Citation Index (SSCI); and CAB abstracts
5. African Index Medicus

We will also search the following conference proceedings:

1. First MIM Pan-African Malaria Conference, Senegal, 6-9 January, 1997
2. Second MIM Pan-African Malaria Conference, South Africa, 15-19 March 1999
3. Third MIM Pan-African Malaria Conference, Tanzania, 17-22 November 2002
4. Fourth MIM Pan-African Malaria Conference, Cameroon, 13-18 November 200;
5. Fifth MIM Pan-African Malaria Conference, Narobi, 2-6 November 2009
6. 59^th^ American Society of Tropical Medicine and Hygiene (ASTMH), Atlanta, 3-7 November 2010

The search terms to be applied can be found in Table S4.

**Study selection**

Studies produced from the search will be independently assessed to determine their eligibility based on whether they fulfil the inclusion criteria highlighted previously. Any disagreements on the eligibility of a study will be resolved through discussion between the authors.

**Data extraction**

Data will be extracted from included studies using an extraction form based on the inclusion criteria highlighted previously. If a situation arises for which there is missing data, or clarity is required, we will contact the study authors in the relevant case.

For dichotomous outcomes, for the ITN and UTN groups, the number of mosquitoes with the event and the total number of mosquitoes will be extracted. For continuous outcomes, we will extract the mean and standard deviation whenever possible. For deterrence, the total number of mosquitoes will be extracted for the ITN and UTN groups.

**Assessment of study rigour**

For hut studies, we generate a set of variables to measure “rigor of implementation”. We have decided to include “rigour of implementation” so that we can assess whether there is any variability in the design and/or execution of the studies. Criteria for rigour will include the following ; procedure for net washing; cleaning of huts; exclusion of male mosquitoes from the data; bioassays and characterisation of resistance in mosquito populations; pre and post study analysis of nets and chemical analysis of nets.

**Risk of bias assessment**

ROB will be assessed separately for each study type. Nine components will be assessed for the hut studies (*i.e.* comparability of mosquitoes of the eligible studies in ITN and UTN huts; collectors blinded; sleepers blinded; incomplete outcome data; measured outcomes described; raw data reported for ITN and UTN groups; ITNs randomly allocated to huts; ITNs rotated; sleepers rotated). Eight components will be assessed for the cone tests and tunnel tests (comparability of mosquitoes in ITN and UTN groups; observers blinded; incomplete outcome data; measured outcomes described; raw data reported for ITN and UTN groups). A judgement of high, low or unclear risk of bias will be made for each component for each study.

**Data analysis**

Data analysis will be performed using Review Manager 5. The analyses will be stratified according to the type of study and also the level of resistance in the mosquito populations: (*i.e.* high, moderate, or low resistance based on phenotypic/genotypic data).

Dichotomous outcomes will be summarised using the risk difference; continuous outcomes will be summarised using mean differences. When a single study compares multiple ITNs, the event rate in the UTN group will be split to ensure that each mosquito is only included in the analysis once.

The results of studies will be pooled using meta-analysis. If heterogeneity is found a random effects approach will be used and in the absence of heterogeneity a fixed effects approach will be taken. Resulting analysis will be presented by forest plots. Heterogeneity will be tested by applying the chi-squared test using a P value of 0.10 to indicate statistical significance, and implementing the I^2^ test statistic with a value of 50% to indicate moderate levels of heterogeneity.

If heterogeneity is detected the possible causes will be explored using subgroup analyses by grouping studies according to the net type (*i.e*. insecticide, dosage, and whether the net was washed or not). Sensitivity analyses will be carried out, such that only hut studies with a low risk of bias for the following components will be included (*i.e*. ITNs randomly allocated to huts, ITNs rotated, sleepers rotated). Reporting biases will be explored using funnel plots.

**References**

1. WHO (2011) World Malaria Report 2011. World Health Organisation.

2. Lengeler C (2004) Insecticide-treated bed nets and curtains for preventing malaria. Cochrane Database Syst Rev: CD000363.

3. WHO (2010) World Malaria Report 2010. World Health Organisation.

4. Ranson H, N'Guessan R, Lines J, Moiroux N, Nkuni Z, et al. (2011) Pyrethroid resistance in African anopheline mosquitoes: what are the implications for malaria control? Trends Parasitol 27: 91-98.

5. Martinez-Torres D, Chandre F, Williamson MS, Darriet F, Berge JB, et al. (1998) Molecular characterization of pyrethroid knockdown resistance (*kdr*) in the major malaria vector *Anopheles gambiae s.s*. Insect Mol Biol 7: 179-184.

6. Ranson H, Jensen B, Vulule JM, Wang X, Hemingway J, et al. (2000) Identification of a point mutation in the voltage-gated sodium channel gene of Kenyan *Anopheles gambiae* associated with resistance to DDT and pyrethroids. Insect Mol Biol 9: 491-497.

7. Hemingway J, Hawkes NJ, McCarroll L, Ranson H (2004) The molecular basis of insecticide resistance in mosquitoes. Insect Biochem Mol Biol 34: 653-665.

8. Djouaka RF, Bakare AA, Coulibaly ON, Akogbeto MC, Ranson H, et al. (2008) Expression of the cytochrome P450s, CYP6P3 and CYP6M2 are significantly elevated in multiple pyrethroid resistant populations of *Anopheles gambiae s.s.* from Southern Benin and Nigeria. BMC Genomics 9: 538.

9. Awolola TS, Oduola OA, Strode C, Koekemoer LL, Brooke B, et al. (2008) Evidence of multiple pyrethroid resistance mechanisms in the malaria vector *Anopheles gambiae sensu stricto* from Nigeria. Trans R Soc Trop Med Hyg.

10. Muller P, Warr E, Stevenson BJ, Pignatelli PM, Morgan JC, et al. (2008) Field-caught permethrin-resistant *Anopheles gambiae* overexpress CYP6P3, a P450 that metabolises pyrethroids. PLoS Genet 4: e1000286.

11. Wood O, Hanrahan S, Coetzee M, Koekemoer L, Brooke B (2010) Cuticle thickening associated with pyrethroid resistance in the major malaria vector *Anopheles funestus.* Parasit Vectors 3: 67.

12. Ranson H, Abdallah H, Badolo A, Guelbeogo WM, Kerah-Hinzoumbe C, et al. (2009) Insecticide resistance in *Anopheles gambiae:* data from the first year of a multi-country study highlight the extent of the problem. Malar J 8: 299.

13. Trape JF, Tall A, Diagne N, Ndiath O, Ly AB, et al. (2011) Malaria morbidity and pyrethroid resistance after the introduction of insecticide-treated bednets and artemisinin-based combination therapies: a longitudinal study. Lancet Infect Dis 11: 925-932.
